# Insights into small-molecule neurotransmitter levels and distribution during tissue regeneration in an ear pinna model in mice

**DOI:** 10.1038/s41598-026-53380-z

**Published:** 2026-05-20

**Authors:** Anfisa Ronda, Rafał Płatek, Paulina Słonimska, Katarzyna Kozłowska-Tylingo, Paweł Sachadyn

**Affiliations:** 1 Laboratory for Regenerative Biotechnology, Gdańsk University of Technology, 80-233 Gdańsk, Poland; 2 Institute of Physical Chemistry, Polish Academy of Sciences (PAS), International Centre for Translational Eye Research - ICTER, Ophthalmic Biology Laboratory, Kasprzaka 44/52 Street, 01-224 Warsaw, Poland; 3 Laboratory of Tissue Engineering and Regenerative Medicine, Division of Clinical Anatomy, Medical University of Gdańsk, 80-211 Gdańsk, Poland; 4 Department of Pharmaceutical Technology and Biochemistry, Gdańsk University of Technology, 80-233 Gdańsk, Poland

**Keywords:** Small-molecule neurotransmitters, Serotonin, Peripheral nervous system, Epigenetics, Zebularine, retinoic acid, Biochemistry, Drug discovery, Neuroscience

## Abstract

Best known for their functions in the nervous system, neurotransmitters also play prominent roles in wound healing. The study analysed the spatiotemporal expression of acetylcholine, dopamine, gamma-aminobutyric acid, serotonin, and its metabolite, 5-hydroxyindoleacetic acid, in regenerating mouse ear pinna during normal healing and during enhanced regeneration induced by the epigenetic inhibitor zebularine and retinoic acid, using HPLC-MS/MS. In terms of time, acetylcholine, dopamine, and gamma-aminobutyric acid showed a gradual decrease during the acute phase on days 3 and 7 post-injury, while serotonin, after a sharp decline on day 3, increased markedly. Zebularine and retinoic acid reduced gamma-aminobutyric acid, acetylcholine, and serotonin in the acute phase. On day 42, over 4 weeks after drug administration and 3 weeks after wound closure, the epigenetic treatment resulted in an elevation in serotonin and a decline in acetylcholine. In spatial terms, the neurotransmitter responses showed similar trends in the wound vicinity and distal tissues, except for a significant increase in serotonin in the wound area and gamma-aminobutyric acid in distal tissues on days 7 and 3, respectively. Decoding the concerted tissue profiles of small-molecule neurotransmitters helps reveal their roles in wound healing and informs the design of regenerative therapies targeting neurotransmitter pathways.

**Supplementary Information:**

The online version contains supplementary material available at 10.1038/s41598-026-53380-z.

## Introduction

The skin, as the body’s external protective barrier, is of exceptional importance. Wound healing and tissue regeneration are vital processes of life. Impaired regenerative capacity not only worsens health quality but can also be life-threatening. Timely skin wound healing is critical to stop bleeding and prevent contamination. Chronic skin wounds are the most prevalent tissue-healing complication, affecting approximately 1–2% of the global population^1,2^. Common causes of impaired skin wound healing include diabetes^3,4^, pressure sores^5,6^ and cancer chemotherapy^7^. The care and treatment of non-healing wounds constitute a significant financial burden for healthcare^8^. Despite the increasing number of patients and multiple research trials, current treatment methods are often ineffective and costly. One of the less-understood yet interesting venues for studying is the role of the nervous system and neurotransmitters in regeneration and wound healing.

Evidence shows that tissue innervation dramatically impacts regenerative capability. A spectacular demonstration of nerve dependence in regeneration is observed in amphibians, where denervation of the stump after amputation abrogates limb regeneration. Also, several studies in mammals point to the involvement of innervation in tissue repair across various models^3,8–12^. A critical role of nerves was demonstrated using surgical denervation in skin and ear pinna hole closure^10,13,14^. Also, diabetic neuropathy has been associated with delayed skin wound healing^3,15^.

The molecular mechanisms underlying nerve involvement in wound-healing and regeneration processes are poorly recognised. It has been found that neurotrophins^16^, neuropeptides^17–19^, and neurohormones^20^, secreted by nerves, contribute to tissue regeneration. Small-molecule neurotransmitters are also considered potential mediators of wound healing and regenerative processes. The roles of serotonin and dopamine^21^ as well as acetylcholine^22^ have been studied in cutaneous wound healing. However, it is worth noting that the primary sources of small-molecule neurotransmitters in wounds are not peripheral nerves, and neurotransmitter receptors are found not only in nerves. Endothelial cells, keratinocytes, fibroblasts, and other cells in wounds express neurotransmitter receptors^22–29^. Notably, nerve fibres are complex neurovascular bundles where larger vessels colocalise with nerves^30^, and fine capillaries integrated with nerve fibres supply them^31^. Therefore, the role of neurotransmitters in tissue regeneration and wound healing extends beyond the nervous system, and neurotransmitter-mediated signalling may play a crucial role in the microenvironment of regenerating tissues.

In this study, we explored small-molecule neurotransmitters during ear pinna regeneration in mice. The ear pinna punch wound emerged as a model of mammalian regeneration first in rabbits^32^. Later, it was discovered that in the MRL mouse strain, 2-mm ear punches close spontaneously without scarring within 4–5 weeks^33^. In contrast, in common-use laboratory strains, 2-mm holes excised in the centre of the ear pinna are considered permanent.

The ear pinna is a complex structure made up of skin, cartilage, muscle layers, and blood and lymphatic vessels that supply the organ^11^. It is also richly innervated^11,30^. Surgical denervation inhibits ear pinna regeneration^10^, highlighting the importance of nerves in this process. Ear pinna wound closure relies on skin growth and can be viewed as a model for cutaneous wound healing^11^. It is also an attractive model for studying mammalian regeneration. While incorporating the immune, circulatory, and nervous systems, it enables convenient tracking of regeneration progress and causes relatively low discomfort to animals^11^. Nevertheless, as with other rodent models, its limitations for human systems should be considered, especially differences at the genetic and immunological levels, and the size of induced lesions.

In a previous study, we developed a pharmacological regenerative model using zebularine and retinoic acid to induce ear pinna hole closure^34^. BALB/c and C57BL/6, widely used laboratory strains, are suitable for testing pro-regenerative drugs because they lack the innate ability to close punched ear holes. Also, BALB/c is known for its docility, an advantage in animal housing and experimentation^11^. The concept of the epigenetic regenerative treatment stems from our previous research on the epigenetic basis of regeneration^35–38^. The concept assumes that zebularine, a nucleoside inhibitor of DNA methyltransferases, reverses the epigenetic silencing of genes essential in regenerative responses, while retinoic acid induces additional transcriptional activation. Notably, an independent laboratory confirmed the pro-regenerative potential of zebularine in ex vivo corneal wound models developed from diabetic patients^39^.

While targeting multiple genes with epigenetic drugs can be effective for inducing regenerative responses that likely depend on multiple genes, the off-target and long-term effects of such therapies require careful investigation. Nevertheless, the existing data seem encouraging. For example, 5-azacitidine and 5-aza-2’-deoxycitidine, approved for the treatment of myeloid malignancies since 2004 and 2006, respectively, like zebularine, act as demethylating agents. Although zebularine did not enter clinical trials, studies show that long-term zebularine administration at doses as high as 400 mg/kg i.p. for 78 days^40^, as well as 7 doses of 1000 mg/kg i.p. for two weeks^34^, demonstrated no harmful effects in mice. Retinoic acid, a metabolite of vitamin A, though it exhibits teratogenic properties, is used not only to treat skin conditions but also acute promyelocytic leukaemia^41^. Finally, regenerative therapies involve stimulating cell dedifferentiation and cell growth, and thus entail the risks associated with such actions.

Tissue regeneration occurs within a complex, evolving niche in which the nervous system and neurotransmitters play a leading role. The expression of neurotransmitters during tissue regeneration and wound healing is most likely precisely coordinated, whereas most available data relate to single molecules. As a result, the field lacks a comprehensive understanding of how their actions are concerted. To address the questions, we performed spatiotemporal profiling of five small-molecule neurotransmitters in ear pinna wounds during normal healing and in tissues regenerating in response to pharmacological treatment. The research focuses on: acetylcholine (ACh), dopamine (DA), gamma-aminobutyric acid (GABA), serotonin (5-HT), and its metabolite 5-hydroxyindoleacetic acid (5-HIAA). The molecules have been previously examined in the context of wound healing, although the data are not abundant. To our knowledge, no studies have determined the concentrations of small-molecule neurotransmitters in healing skin wounds or regenerating tissues.

## Results

### Development of neurotransmitters determination

As no established method for the simultaneous determination of DA, ACh, GABA, 5-HT, and 5-HIAA in solid tissues was available, we developed an original protocol for analyte extraction and HPLC-MS/MS detection. The results of HPLC-MS/MS optimisation, sample preparation, extraction recovery, and linearity measurements are detailed in Supplemental File 1.

### Spatiotemporal expression of neurotransmitters in regenerating ear pinna

**Fig. 1:**
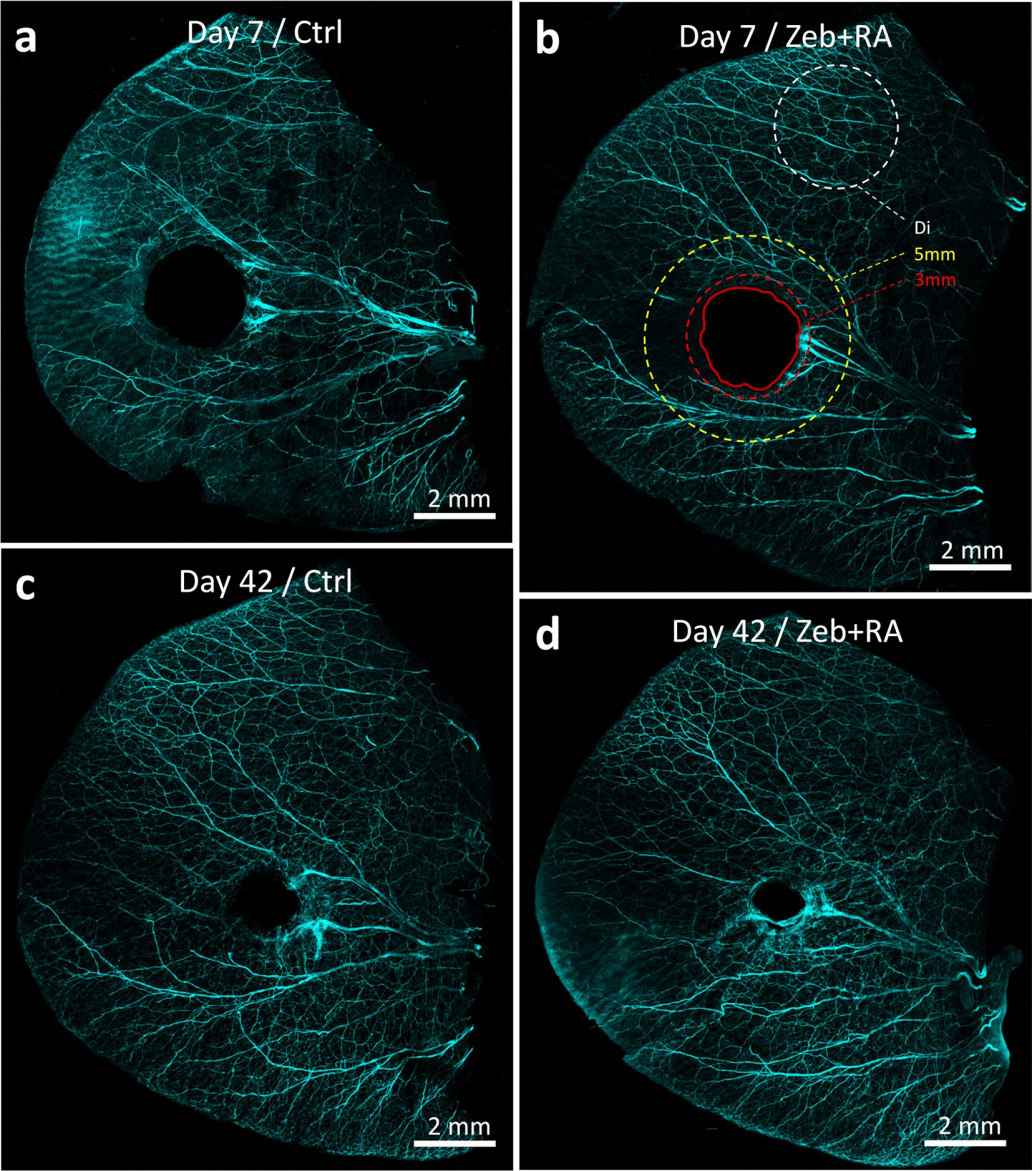
Locations of tissues collected for neurotransmitter analyses superimposed on images of nerve networks in the regenerating ear pinna. Confocal images of the outer aspect of the mouse ear pinna after whole mount immunofluorescent staining for a neuron-specific anti-β-III-Tubulin depicting peripheral nerves (turquoise pseudocolour) from control (Ctrl) mice (**a** and **c**) or zebularine (Zeb) + retinoic acid (RA) treated mice (**b** and **d**) at day 7 (**a, b**) and day 42 (**c, d**) post-injury. (**b**) Solid claret-coloured outline marks the wound margin; red, yellow and white dashed outlines mark 3mm, 5mm and distal (Di) compartments of tissue sampled in the study.

**Fig. 2:**
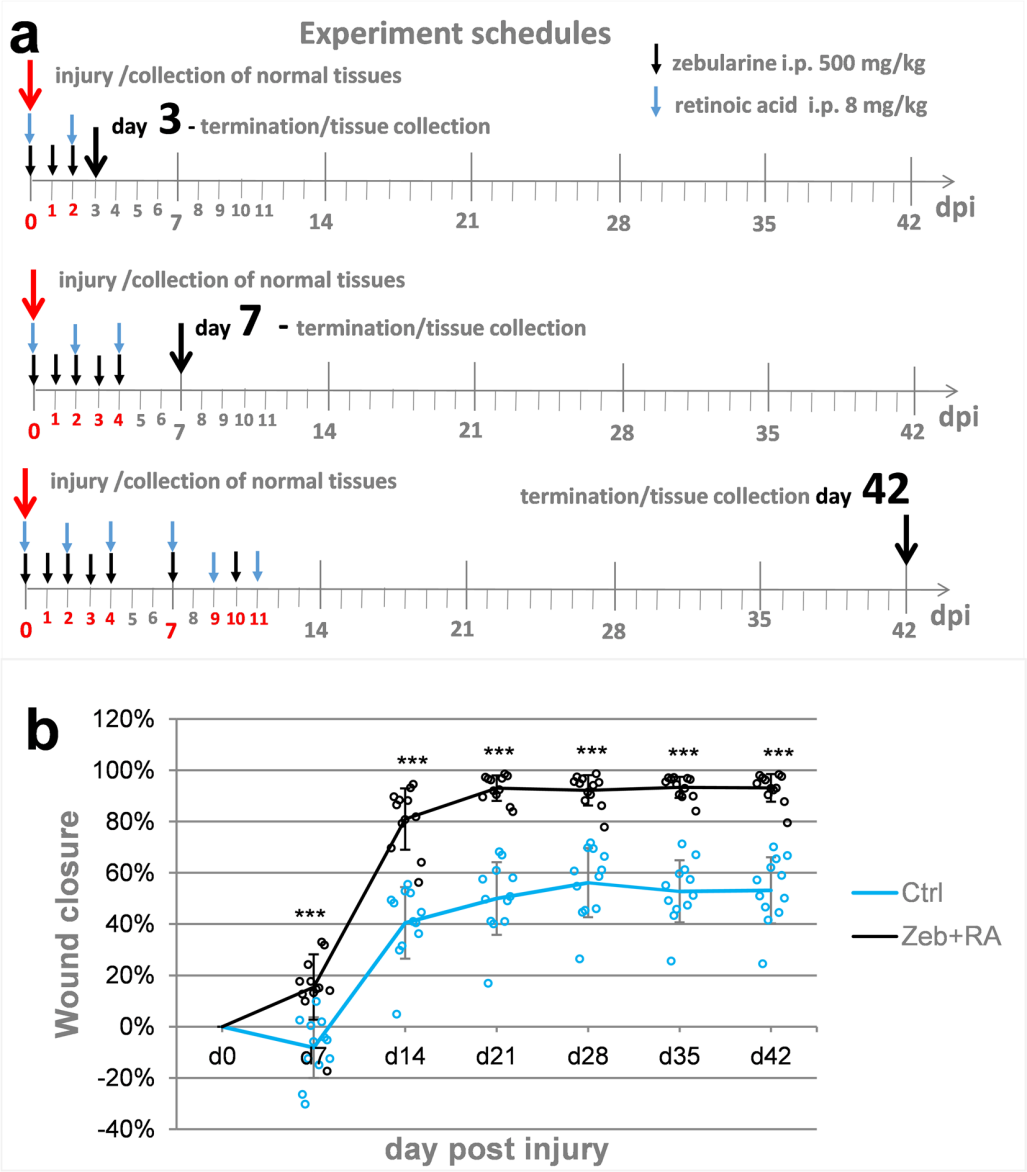
Regenerative ear pinna wound closure induced by zebularine and retinoic acid. (**a**) Schedule of zebularine and retinoic acid administration and tissue collection at 3, 7 and 42 days post-injury; (**b**) progress of ear pinna wound closure in 6 Ctrl mice (n = 12 wounds) and 6 Zeb+RA-treated mice (n = 12 wounds). The Mann-Whitney *U* test was used for comparisons between treatments. Error bars represent SD. Three asterisks indicate statistical significance of *p* < 0.001.

To shed more light on the activity of small-molecule neurotransmitters during tissue regeneration, we aimed to characterise their spatiotemporal concentrations in the ear pinna after injury. To achieve this, we used high-performance liquid chromatography-tandem mass spectrometry (HPLC-MS/MS) to determine the concentrations of five neurotransmitters: (i) γ-aminobutyric acid (GABA); (ii) acetylcholine (ACh); (iii) dopamine (DA); (iv) serotonin (5-HT), and (v) serotonin’s metabolite—5-hydroxyindoleacetic acid (5-HIAA) in regenerating ear pinna tissues in mice. To examine the neurotransmitter levels at different distances from the injury, we collected tissues from four ear pinna locations (Fig. 1b): (i) the controls designated as normal tissue (Nt), i.e., 2-mm circles from the central part of the auricle excised at the wounding and placed immediately in the liquid nitrogen; (ii) 3-mm rings immediate to the wounds (3mm); (iii) 5-mm rings surrounding the wound and regeneration area (5mm), and iv) 3-mm discs excised from the auricle part distant to the wound (Di). To analyse neurotransmitter levels’ time changes we collected the samples at four time points: (i) day 0, i.e., normal tissue represented by the 2-mm ear pinna circles excised at the injury; (ii) day 3 and (iii) day 7 falling into the acute (inflammatory) phase and showing limited if any tissue growth (Fig. 2b) but critical for the regeneration outcomes^11^ and iv) and day 42 post-injury, corresponding to the remodelling phase, after wound closure is completed. In parallel, we determined the neurotransmitter levels in ear pinna during enhanced regeneration induced by systemic administration of zebularine and retinoic acid (Zeb+RA) (Fig. 2a). The epigenetic treatment significantly improved and accelerated ear pinna hole closure (Fig. 2b). Representative whole-mount confocal images demonstrate nerve fibre networks formed around the wounds in both treatment and control mice (Fig. 1a–d) on days 7 and 42 post-injury. The spatiotemporal profiles of the neurotransmitter under study are described below.

**Fig. 3:**
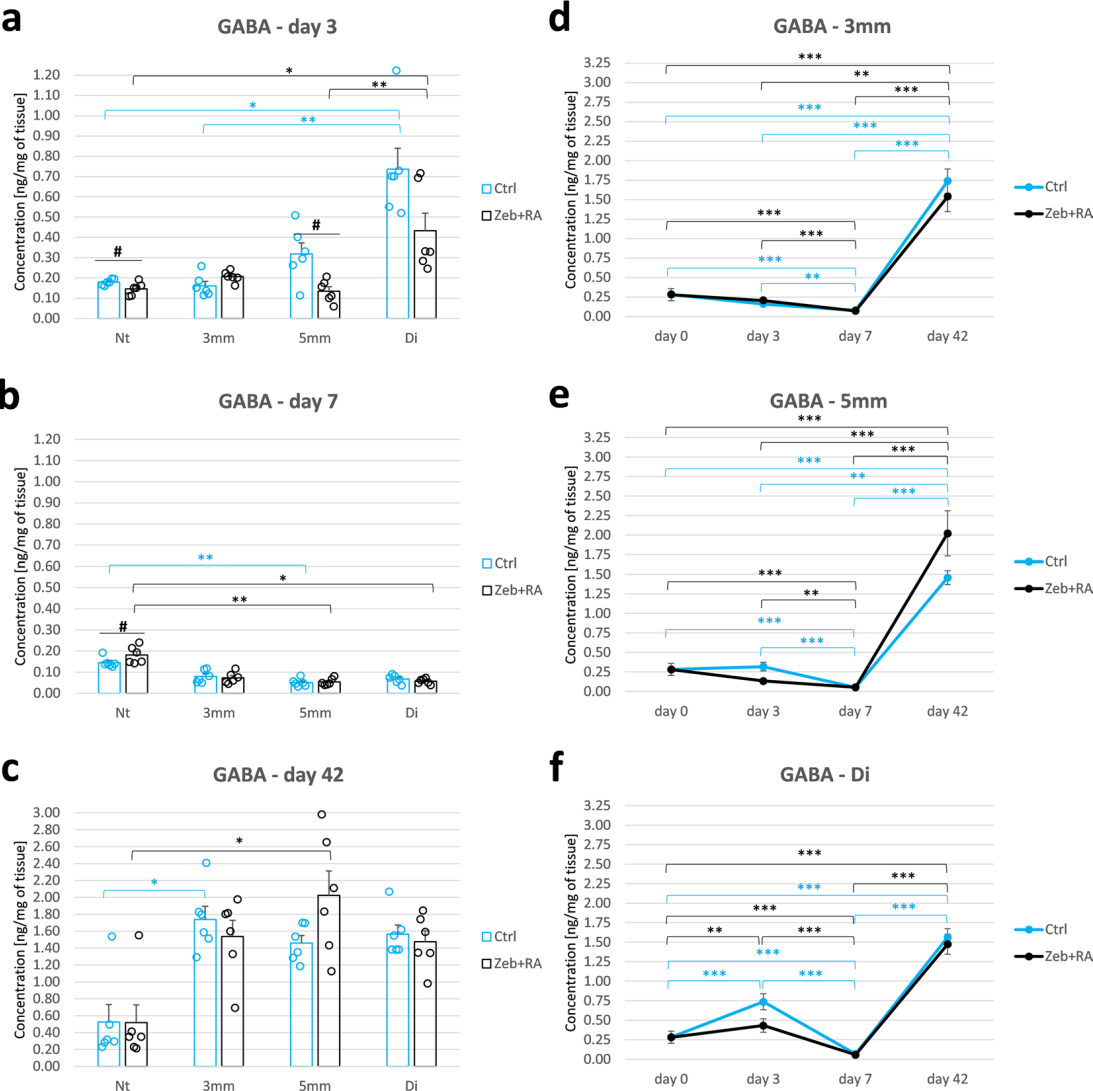
Concentrations of γ-aminobutyric acid (GABA) in mouse ear pinna tissue. Nt/day 0 refers to 2-mm discs of normal tissue collected at day 0; 3mm, 5mm, and Di compartments refer to tissues collected on days 3, 7 or 42 post-injury, specifically from 3- and 5-mm rings surrounding the wound and a site distant from the wound (Di). Zeb+RA (n = 6)-group subjected to pharmacological treatment with zebularine (Zeb) and retinoic acid (RA); Ctrl (n = 6)-control group injected with the vehicle only. (**a–c**) comparison of GABA concentrations between tissue compartments within each time point and between treatments; Nt denotes normal tissues collected at individual time points (n = 6). (**d–f**) comparison of GABA concentrations between time points: days 0 and 3, 7, 42 post-injury within each tissue compartment (day 0 denotes assembled data from 3 experimental groups for Ctrl (*n* = 18) and for Zeb+RA (n = 18). For between-compartments comparisons of paired samples (**a–c**), the Friedman’s test was used. For between-treatment comparisons (**a–c**), the Mann-Whitney U-test was applied. For between-time-point comparisons (**d–f**), the Kruskal–Wallis test with multiple pairwise comparisons using the Conover-Iman post-hoc test was employed. Error bars represent SEM. One, two, or three asterisks indicate statistical significance of *p* < 0.05, *p* < 0.01, or *p* < 0.001, respectively. One hashtag indicates statistical significance (*p* < 0.05). *p*-values and other details of statistics are listed in Supplemental File 3.

**Fig. 4:**
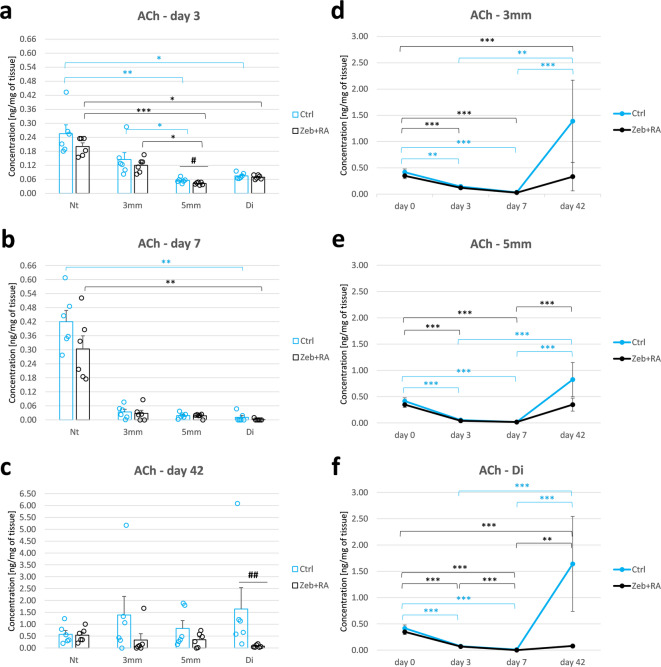
Concentrations of acetylcholine (ACh) in mouse ear pinna tissue: Nt/day 0 refers to 2-mm discs of normal tissue collected at day 0; 3mm, 5mm, and Di compartments refer to tissues collected on days 3, 7 or 42 post-injury, specifically from 3- and 5-mm rings surrounding the wound and a site distant from the wound (Di). Zeb+RA (*n* = 6)-group subjected to pharmacological treatment with zebularine (Zeb) and retinoic acid (RA); Ctrl (*n* = 6)-control group injected with the vehicle only. **a–c**) comparison of ACh concentrations between tissue compartments within each time point and between treatments; Nt denotes normal tissues collected at individual time points (n = 6). **d–f**) comparison of ACh concentrations between time points: days 0 and 3, 7, 42 post-injury within each tissue compartment. For between-compartments comparisons of paired samples (**a–c**), the Friedman’s test was used. For between-treatment comparisons (**a–c**), the Mann–Whitney U-test was applied. For between-time-point comparisons (**d–f**), the Kruskal–Wallis test with multiple pairwise comparisons using the Conover-Iman post-hoc test was employed. Error bars represent SEM. One, two, or three asterisks indicate statistical significance of *p* < 0.05, *p* < 0.01, or *p* < 0.001, respectively. One or two hashtags indicate statistical significance of *p* < 0.05 or *p* < 0.01, respectively. *p*-values and other details of statistics are listed in Supplemental File 3.

**Fig. 5:**
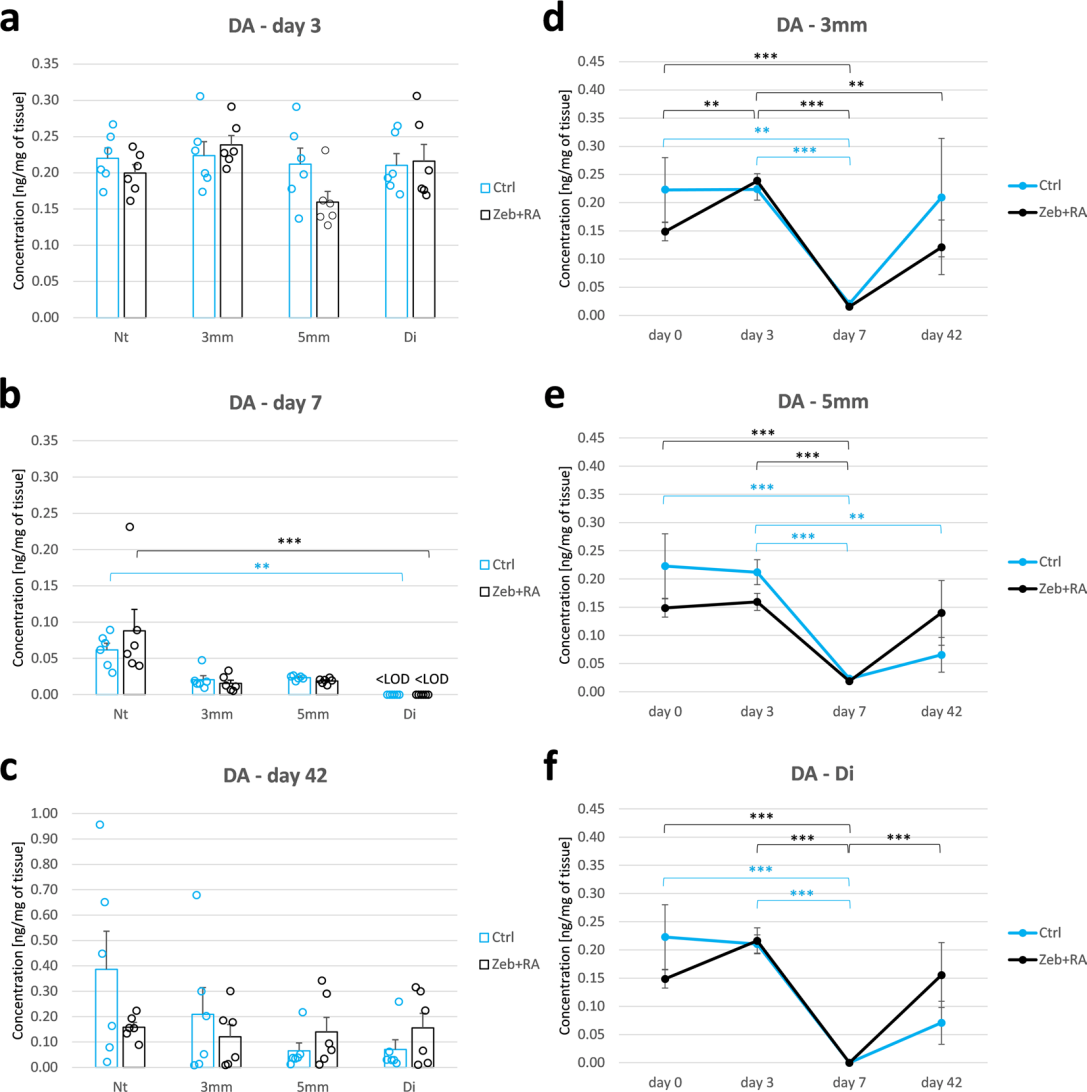
Concentrations of dopamine (DA) in mouse ear pinna tissue. Nt/day 0 refers to 2-mm discs of normal tissue collected at day 0; 3mm, 5mm, and Di compartments refer to tissues collected on days 3, 7 or 42 post-injury, specifically from 3 mm to 5- mm rings surrounding the wound and a site distant from the wound (Di). Zeb+RA (n = 6)-group subjected to pharmacological treatment with zebularine (Zeb) and retinoic acid (RA); Ctrl (n = 6) - control group injected with the vehicle only. **a–c**) comparison of DA concentrations between tissue compartments within each time point and between treatments; Nt denotes normal tissues collected at individual time points (n = 6). **d–f**) comparison of DA concentrations between time points: days 0 and 3, 7, 42 post-injury within each tissue compartment (day 0 denotes assembled data from 3 experimental groups for Ctrl (n = 18) and for Zeb+RA (n = 18). For between-compartments comparisons of paired samples (**a–c**), the Friedman’s test was used. For between-treatment comparisons (**a–c**), the Mann–Whitney U-test was applied. For between-time-point comparisons (**d–f**), the Kruskal–Wallis test with multiple pairwise comparisons using the Conover-Iman post-hoc test was employed. Error bars represent SEM. Two or three asterisks indicate statistical significance of *p* < 0.01 or *p* < 0.001, respectively. *p*-values and other details of statistics are listed in Supplemental File 3.

**Fig. 6:**
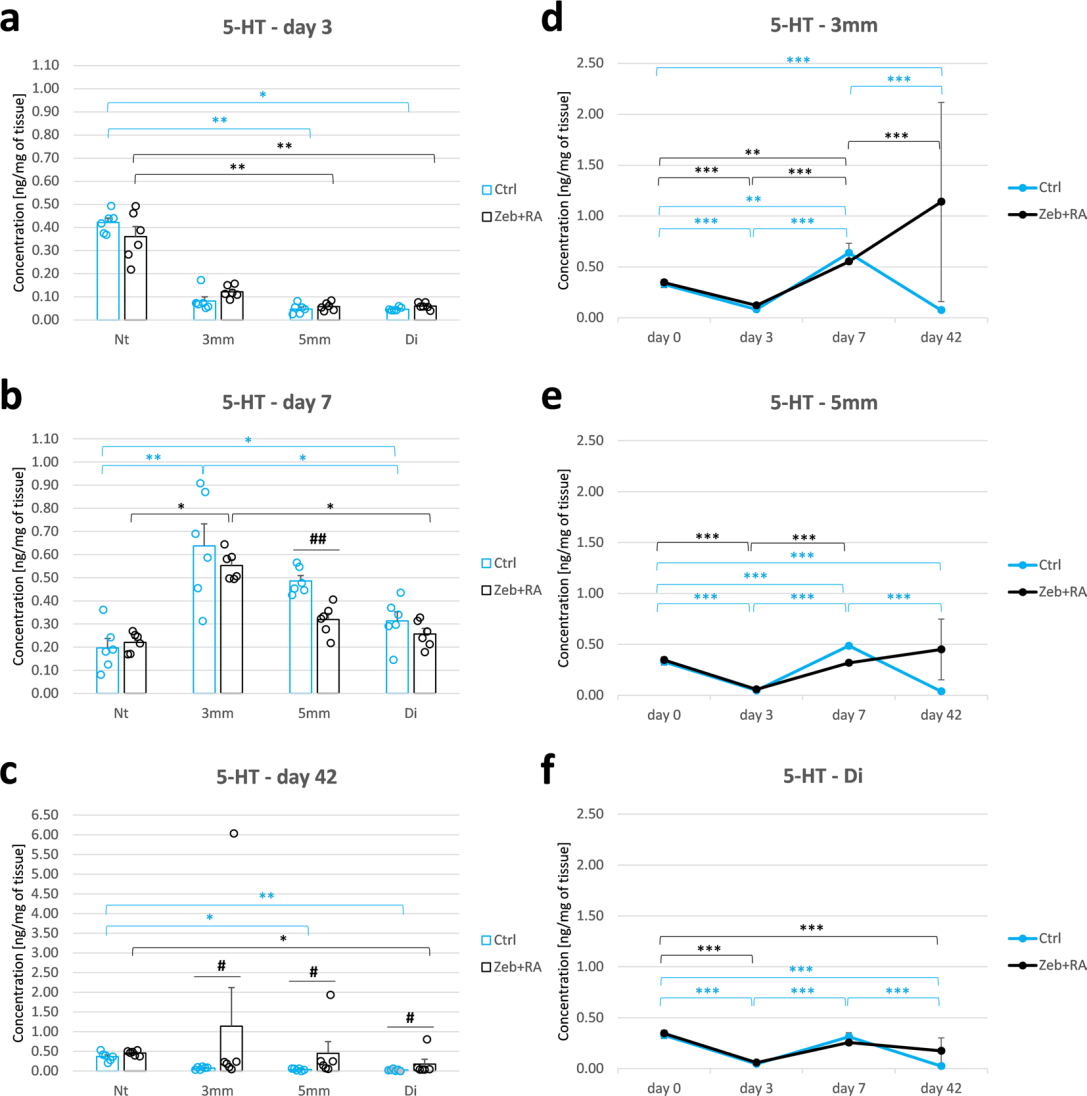
Concentrations of serotonin (5-HT) in mouse ear pinna tissue. Nt/day 0 refers to 2-mm discs of normal tissue collected at day 0; 3mm, 5mm, and Di compartments refer to tissues collected on days 3, 7 or 42 post-injury, specifically from 3- and 5-mm rings surrounding the wound and a site distant from the wound (Di). Zeb+RA (n = 6)-group subjected to pharmacological treatment with zebularine (Zeb) and retinoic acid (RA); Ctrl (n = 6)-control group injected with the vehicle only. (**a–c**) comparison of 5-HT concentrations between tissue compartments within each time point and between treatments; Nt denotes normal tissues collected at individual time points (n = 6). (**d–f**) comparison of 5-HT concentrations between time points: days 0 and 3, 7, 42 post-injury within each tissue compartment (day 0 denotes assembled data from 3 experimental groups for Ctrl (n = 18) and for Zeb+RA (n = 18). For between-compartments comparisons of paired samples (**a–c**), the Friedman’s test was used. For between-treatment comparisons (**a–c**), the Mann–Whitney U-test was applied. For between-time-point comparisons (**d–f**), the Kruskal–Wallis test with multiple pairwise comparisons using the Conover-Iman post-hoc test was employed. Error bars represent SEM. One, two, or three asterisks indicate statistical significance of *p* < 0.05, *p* < 0.01, or *p* < 0.001, respectively. One or two hashtags indicate statistical significance of *p* < 0.05 or *p* < 0 0.01, respectively. *p*-values and other details of statistics are listed in Supplemental File 3.

**Fig. 7:**
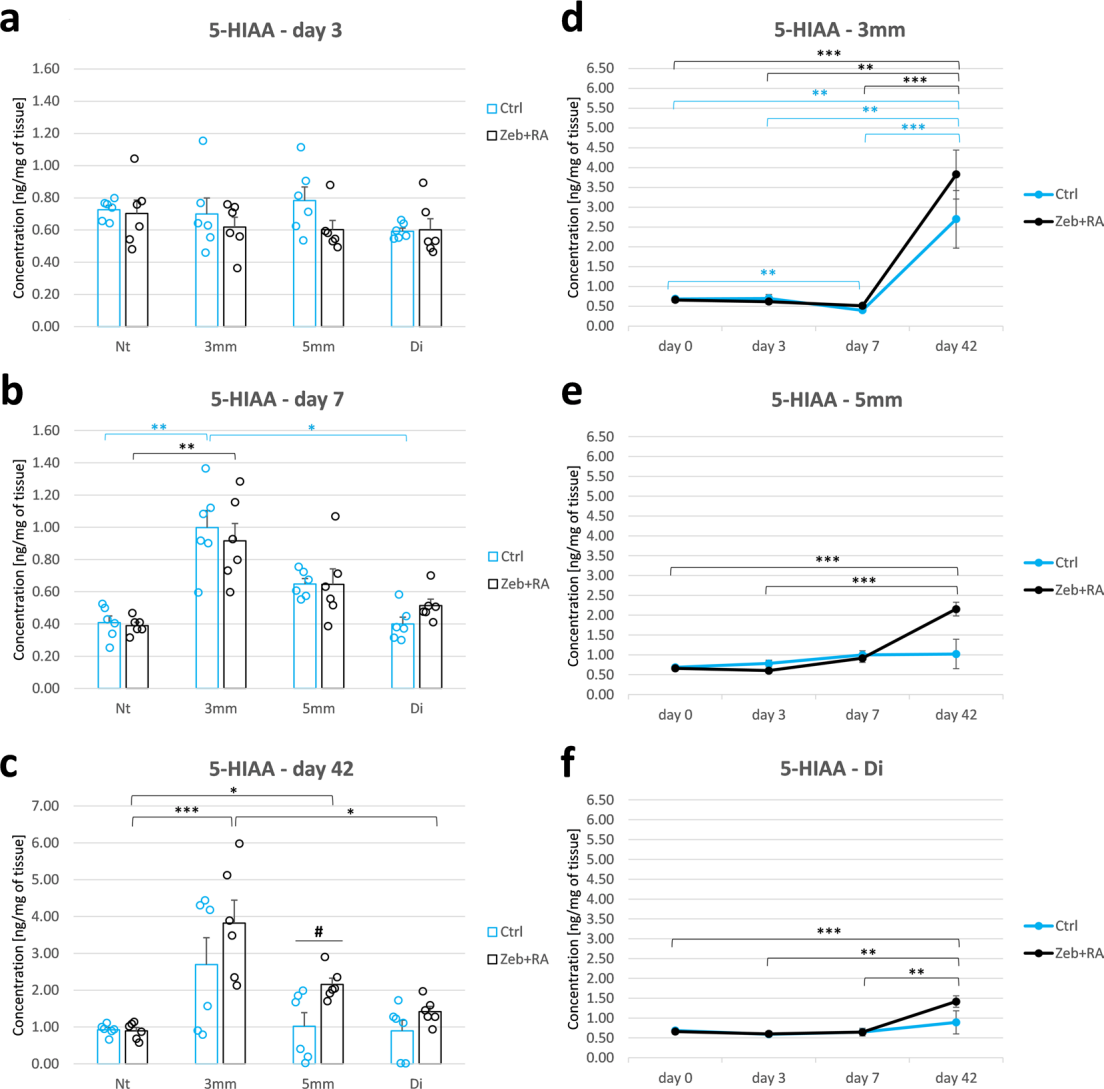
Concentrations of 5-hydroxyindoleacetic acid (5-HIAA) in mouse ear pinna tissue. Nt/day 0 refers to 2-mm discs of normal tissue collected at day 0; 3mm, 5mm, and Di compartments refer to tissues collected on days 3, 7 or 42 post-injury, specifically from 3- and 5-mm rings surrounding the wound and a site distant from the wound (Di). Zeb+RA (n = 6)-group subjected to pharmacological treatment with zebularine (Zeb) and retinoic acid (RA); Ctrl (n = 6) - control group injected with the vehicle only. (**a–c**) comparison of 5-HIAA concentrations between tissue compartments within each time point and between treatments; Nt denotes normal tissues collected at individual time points (n = 6). (**d–f**) comparison of 5-HIAA concentrations between time points: days 0 and 3, 7, 42 post-injury within each tissue compartment (day 0 denotes assembled data from 3 experimental groups for Ctrl (n = 18) and for Zeb+RA (n = 18). For between-compartments comparisons of paired samples (**a–c**), the Friedman’s test was used. For between-treatment comparisons (**a–c**), the Mann–Whitney U-test was used. For between-time-point comparisons (**d–f**), the Kruskal–Wallis test with multiple pairwise comparisons using the Conover-Iman post-hoc test was employed. Error bars represent SEM. One, two, or three asterisks indicate statistical significance of *p* < 0.05, *p* < 0.01, or *p* < 0.001, respectively. One hashtag indicates statistical significance (*p* < 0.05). *p*-values and other details of statistics are listed in Supplemental File 3.

Of note, neurotransmitter determination required tissue collection after euthanasia. Therefore, we assigned separate groups of animals to each time point. For temporal change plots, we combined day 0 samples from all three groups representing day 3, day 7 and day42. In this way, we compared day 0 with days 3, 7, and 42 (Figs. 3, 4, 5, 6 and 7d–f). For spatial comparisons, each time point was analysed separately, so normal tissues (Nt) and all compartments (3 mm, 5 mm, Di) were obtained from the same set of ear pinnae for each plot (Figs. 3, 4, 5, 6 and 7a–c).

### GABA

In function of distance from the injury (Fig. 3a-c), GABA levels at day 3 demonstrated an increase towards the edge of the ear pinna, with the highest levels in Di in both Ctrl and Zeb+RA. On days 7 and 42 after the injury, GABA levels were uniformly distributed between 3mm, 5mm, and Di, showing a decrease on day 7 and an increase on day 42 compared to Nt (Figs. 3b and 5c). Comparisons over time (Fig. 3d–f) showed a similar profile of GABA levels within 3 mm, 5mm, and Di, with a significant decrease at day 7 and a sharp increase at day 42 compared to day 0 in both Zeb+RA and Ctrl (Fig. 3d–f). Treatment-wise, there were no marked differences, except that Zeb+RA significantly lowered GABA levels in the acute phase at day 3- in 5mm rings, compared to the Ctrl group (Fig. 3a).

### ACh

In function of distance from the injury (Fig. 4a–c), ACh levels at day 3, opposite to GABA, dropped towards the edge of the ear pinna with the lowest levels in 5mm and Di compared to Nt and 3mm in both Ctrl and Zeb+RA. Similarly to GABA, ACh at day 7 was uniformly distributed between 3 mm, 5mm and Di compartments with overall tendency and a significant decrease in Di compared to Nt (Fig. 4b). There were no significant differences in ACh between compartments on day 42 (Fig. 4c). Comparisons over time showed a similar profile of ACh levels within 3 mm, 5mm and Di (Fig. 4d–f), with a significant decrease at days 3 and 7 post-injury relative to day 0 and a subsequent uplift at day 42 towards the levels from day 0. This profile resembled that of GABA, except that ACh in Zeb+RA-treated mice did not exceed the pre-injury day 0 levels at day 42. Treatment comparisons showed that Zeb+RA significantly lowered ACh levels at day 3- in 5- mm rings, similar to GABA (Fig. 4a). Additionally, at day 42, Zeb+RA showed a general tendency toward a decrease in ACh, with a significant drop in Di compared to Ctrl (Fig. 4c).

### DA

In function of distance from the injury (Fig. 5a–c), DA showed homogenous levels between compartments after injury, not different from Nt in both Zeb+RA and Ctrl groups, except on day 7, with Di below the detection level (Fig. 5b). Comparisons over time (Fig. 5d–f) showed a similar profile of DA levels within 3mm, 5mm and Di, with a significant decrease at day 7 post-injury compared to day 0 and day 3, with subsequent rises at day 42 towards the control levels in treated and control groups. Figure 5d–f). Treatment comparisons showed no effect of Zeb+RA application on DA (Fig. 5a–c).

### 5-HT

In function of distance from the injury (Fig. 6a–c), 5-HT levels at day 3 were homogeneous between the 3mm, 5mm, and Di compartments but lower compared to Nt, with significant decreases for 5mm and Di. At day 7, 5-HT levels significantly increased in proximity to the lesion compared to Nt and Di in both Zeb+RA and Ctrl. On day 42, 5-HT profiles markedly decreased compared to Nt in Ctrl, while the opposite tendency was displayed in Zeb+RA, especially in proximity to injury. Comparisons over time (Fig. 6d–f) among groups showed similar profiles of 5-HT within 3mm, 5mm, and Di. In contrast to other neurotransmitters under study, 5-HT levels dropped earlier, reaching their lowest point on day 3 (Fig. 6d–f). Notably, there is a further increase in 5-HT in the proximity of the wound margin (3mm) towards day 42 in Zeb+RA (Fig. 6d) but not in Ctrl, where 5-HT levels drop once again below those of day 0 (Fig. 6d–f). Treatment comparisons revealed a significant reduction in 5-HT in the acute phase, at day 7 in 5mm (Fig. 6b), and remarkable increases in all compartments after the injury (3mm, 5mm and Di) in the remodelling phase (day 42) (Fig. 6c).

### HIAA

In the function of distance from the injury (Fig. 7a–c), 5-HIAA levels on day 3 stayed unchanged between the 3mm, 5mm, and Di compartments and also compared to Nt (Fig. 7a). At day 7, 5-HIAA levels were similarly increased as for 5-HT - in proximity to the lesion (3mm) compared to Nt (Fig. 7b). At day 42, 5-HIAA levels were still significantly elevated in the proximity of the wound (3mm and 5mm) only in the Zeb+RA group. Comparisons over time (Fig. 7d–f) showed similar 5-HIAA profiles within 3mm, 5mm and Di with no changes within 7 days after the injury and a subsequent increase at day 42, especially pronounced after Zeb+RA treatment (Fig. 7d–f). Treatment comparisons revealed that, like for 5-HT, Zeb+RA increased 5-HIAA levels compared to Ctrl in the remodelling phase on day 42 (Fig. 7c); however, the change was significant only at 5mm.

### Expression of fibroblast activation marker genes

Given the link between 5-HT and fibroblast activity, we examined molecular markers of fibroblast and myofibroblast induction in regenerating ear pinnae: *Acta2*, which encodes α-SMA, *a*nd *Ccn2*, which encodes connective tissue growth factor. We recorded a transient elevation of *Acta2* expression in the acute phase at day 7 in the direct wound vicinity. The most spectacular, however, were the increases of both *Acta2* and *Ccn2* expression following the epigenetic treatment, on day 42, which falls within the remodelling phase (Supplemental Fig. S1).

## Discussion

The neurotransmitters GABA, ACh, DA, 5-HT, and 5-HIAA, primarily known for their roles in the central nervous system, are also investigated as regulatory factors involved in cutaneous wound healing. However, there is relatively little work on this topic. As small endogenous molecules, these neurotransmitters may be attractive candidates for improving skin wound healing. To determine the concentrations of neurotransmitters in tissues during wound healing, we selected a model of regenerating ear pinna in mice.

We focused the analyses on the early post-injury (acute) phase, specifically days 3 and 7, due to the critical role of the inflammatory phase in wound healing. For comparison, we examined normal tissue (Nt) and the samples collected on day 42 post-injury, 3 weeks after wound closure had stopped (Fig. 2b). In parallel, we conducted the analyses in mice receiving pharmacological pro-regenerative treatment. Based on an epigenetic derepression strategy, the treatment involved the combined administration of zebularine and retinoic acid. In our previous study, we demonstrated that retinoic acid and zebularine exhibited synergistic effects, resulting in accelerated, near-complete ear punch wound closure that did not occur in control mice^34^. Notably, restoration of tissue architecture accompanied wound closure. In the ear punch wound experiments performed in this study, we also observed nearly perfect ear pinna hole closure following the epigenetic treatment (Fig. 2b). Thus, we examined the neurotransmitter responses to wounding in both normal physiological wound healing and in a model of enhanced tissue regeneration.

Mice underwent isoflurane anaesthesia prior to injury and were euthanised in a carbon dioxide chamber before tissue collection from wounded ear pinnae. Although the effects of these procedures on peripheral neurotransmitters are not well characterised, they may represent potential confounding factors. Nonetheless, we want to stress that identical experimental conditions were applied to both treatment and control mice. Isoflurane interference seems less likely, as anaesthesia was administered several days before tissue collection. Euthanasia, in turn, was performed immediately prior to tissue collection; however, the use of a gradually increasing carbon dioxide concentration is expected to prevent pain and thus its impact on neurotransmitters.

We determined significant changes in neurotransmitter concentration in ear pinna tissues over time post-injury, with distance from the wound, and in response to the epigenetic pharmacological treatment. Below, we discuss the results in the context of existing literature data and their impact on wound healing and tissue regeneration.

### 5-HT

Of the five neurotransmitters analysed in the study, serotonin (5-HT) attracted our attention owing to substantially higher levels after Zeb+RA treatment compared to control mice (Ctrl) on day 42 post-injury, that is, over 4 weeks after drug administration. Also noteworthy was a 34% significant decrease in Zeb+RA-treated mice on day 7 post-injury in 5-mm rings surrounding, but not immediately contacting the wound.

Serotonin or 5-hydroxytryptamine (5-HT) is synthesised from L-tryptophan by enterochromaffin cells (EC) localised in the mucosa of the gastrointestinal tract and neurons of the central and autonomic nervous systems—raphe nuclei of the brainstem and autonomic neurons forming neuromuscular junctions respectively^42–44^. The brain’s serotonergic neurons are known to produce only a minor fraction (1–2%) of the body’s total 5-HT; the vast majority, approximately 90%, originates from enterochromaffin cells (EC)^45,46^. The gut 5-HT synthesised by ECs is broadly taken up by platelets and mast cells via the 5-HT transporter, constituting the primary source of 5-HT for peripheral tissues^47–49^. 5-HT is hypothesised to participate in wound healing^50^ by regulating inflammation and tissue repair through a large set of 15 receptors grouped into seven families: 5-HTR_1−7_^51^. Several immune cells involved in the healing process express distinct sets of 5-HT receptors. The literature indicates that 5-HT primarily influences wound healing through immunomodulation and the activation of fibroblasts. 5-HT modulates immune responses by enhancing or suppressing activities in lymphocytes (B and T cells), natural killer (NK) cells, monocytes/macrophages, and dendritic cells, thereby fine-tuning their functional outputs^50^. Upon injury, 5-HT released from platelets induces coagulation and haemostasis through local constriction of the microvasculature^48,52^. 5-HT at the site of injury promotes inflammation by stimulating mast cell migration and adhesion through the 5-HT_1A_ receptor^53^ and eosinophil migration through 5-HT_2A_ receptor activation^54^. Acting via 5-HT_3_, 5-HT_4_, and 5-HT_7_ receptors, 5-HT increases IL-6 expression in mature dendritic cells^25,55^, further amplifying the positive loop of inflammation during the inflammatory phase of wound healing^48,56^. On the other hand, activation of the 5-HT_7_ receptor has been shown to induce early T-cell proliferation and activation^27,50^. During wound repair, monocytes/macrophages are pivotal in driving inflammation^57,58^. Localised injury cues trigger resident macrophages, whereas monocytes extravasate from the bloodstream into the injury microenvironment in response to chemotactic signals and, at the site of injury, acquire the pro-inflammatory M1 phenotype^58,59^. In this state, macrophages are engaged in clearing dead cells, mobilising stem/progenitor cells, and chemotaxis of other immune cells to the lesion site^56,60^. Macrophages dynamically transition from a pro-inflammatory state to a reparative M2 phenotype over time, acquiring immunosuppressive properties that facilitate tissue regeneration by stimulating proliferation, extracellular matrix remodelling, and angiogenesis^56,61^. Mice lacking the 5-HTR_1A_ presented impaired wound healing, characterised by increased macrophage accumulation at the injury site and a prolonged inflammatory phase^62^. 5-HT was also shown to regulate macrophage polarization through their 5-HT_2B_ and 5-HT_7_ receptors, enhancing M2-associated genes and suppressing M1-related markers. While the suppression of pro-inflammatory cytokines relies on 5-HT_7_ activation, both receptors collaborate to induce M2-associated genetic reprogramming^50,63^.

There is substantial data connecting increased 5-HT levels with tissue fibrosis^64^, as observed in cardiac valves, lungs, and skin in subjects with carcinoid neuroendocrine tumours that secrete vast quantities of 5-HT. Similarly, persistent 5-HT signalling disrupts healing processes during chronic tissue injury, driving maladaptive repair mechanisms that culminate in fibrosis and diminished regenerative capacity^48^. A spectacular demonstration of the 5-HT pro-fibrotic effects is the experiment involving subcutaneous injections of high 5-HT doses in rats, which resulted in scarring^65^. However, the above-described outcomes are related to extreme and persistent 5-HT increases, whereas data suggest that 5-HT has beneficial effects on cutaneous wound healing. Specifically, 5-HT enhances macrophage recruitment during the inflammatory phase of wound healing, stimulates keratinocyte migration and fibroblast activity in the growth phase, and supports tissue remodelling through collagen production and stabilisation of new vessels^66^. Sadiq et al. reported impaired excisional skin wound healing in mice lacking the 5-HT_1A_ receptor, and signs of improved healing in wounds treated topically with a 5-HT_1A_ receptor agonist, (R)-(+)-8-Hydroxy-DPAT hydrobromide^62^.

Furthermore, topical treatment of excisional skin wounds in diabetic mice with 0.2% fluoxetine, a selective serotonin reuptake inhibitor, promoted enhanced wound healing^67^. Interestingly, as low as 540 ng/L (0.000054%), fluoxetine concentrations were demonstrated to improve wound closure in an in vitro scratch assay^68^. Fluoxetine, when administered in drinking water, promoted regeneration and muscle function in mice after notexin-induced injury to the *Tibialis anterior* muscle^69^. The recent findings on fluoxetine’s regenerative actions are exciting, but the impact of fluoxetine administration on peripheral 5-HT remains unclear. Both increased and decreased 5-HT, as well as marked differences in platelet and plasma levels, have been reported in response to fluoxetine^70^. Topical application of other serotonin-modulating medications is investigated in the management of chronic wounds^66^.

The effect of Zeb+RA treatment on 5-HT in the regenerating ear pinnae was significant on day 7 post-injury. Still, the change was moderate (Fig. 6b). In contrast, it was remarkable in the regenerated and noninjured parts of the ear pinnae on day 42 post-injury, corresponding to the remodelling phase. Compared with normal pre-injury tissues, 5-HT levels were strikingly lower in control mice. In contrast, in Zeb+RA-treated animals, there were marked increases in 5-HT in the immediate vicinity of the wound (3mm) (Fig. 6c).

Elevated 5-HT levels can stimulate fibroblast activity. The induction of *Acta2* and *Ccn2* genes (Supplemental Fig. S1) was consistent with the 5-HT levels. Persistent and excessive Acta2 (α-SMA) expression is a hallmark of pathological fibrosis. However, transient activity of α-SMA-expressing differentiated myofibroblasts is essential to attain the proper tensile strength of connective tissue in the skin^71^ and vessels^72^. Moreover, genetic depletion of α-SMA-positive myofibroblasts results in a plethora of wound healing defects^73^. Considering that histological examinations reveal no excessive scarring in the regenerated ear pinna after Zeb+RA treatment^34,74^, and zebularine has been reported to attenuate tubulointerstitial fibrosis^75^, the elevated levels of 5-HT and *Acta2* we observe are unlikely to reflect pathological fibrosis. Conversely, they may be associated with the restoration of tissue integrity, specifically the formation of nerve and vessel networks we observe in the regenerating ear pinnae following the epigenetic treatment^11^. At the same time, the decrease in 5-HT may reflect the earlier termination of wound healing in the controls.

### HIAA

5-HT activity depends on its rate of synthesis, release, and metabolism. After ear pinna injury, we analysed the levels of its primary metabolite 5-HIAA. In agreement with the increased 5-HT concentrations observed in the chronic phase, 5-HIAA levels were also elevated at day 42 compared to earlier time points in both Ctrl and Zeb+RA-treated groups. However, only after Zeb+ RA treatment did the increased 5-HIAA levels extend outside the 3mm compartment (Fig. 7d–f).

### DA

Research demonstrates that dopamine plays a dual role in the regeneration process, acting both as an agonist and an antagonist of wound healing, depending on receptor-specific effects. D2 receptor activation impedes wound healing in diabetic models and suppresses tumour angiogenesis by downregulating VEGF activity - a key driver of blood vessel formation. Pharmacological blockade of D2 receptors counteracts this suppression, enhancing the expression of HoxD3 and integrin α5β1, molecules critical for epithelial regeneration ^76,77^. Conversely, D1 receptor stimulation promotes healing by upregulating VEGFA levels, thereby supporting angiogenic processes^23^. This dual regulatory mechanism arises from opposing signalling pathways: D1-mediated CREB phosphorylation enhances VEGFA production, while D2 receptor activation suppresses vascular permeability factor/VEGF via HoxD3 phosphorylation. DA involvement in wound-healing regulation prompted testing dressings incorporating dopamine to improve wound closure^78^. Our analysis of DA concentrations in the auricular tissues showed no significant effect of the Zeb+RA treatment, and comparisons over time showed similar profiles of treated and control mice. Notably, DA decreases on day 7 post-injury in both control and treatment mice (Fig. 5d–f).

### ACh

Acetylcholine is produced in neurons acting at neuronal and neuromuscular junctions, as well as in non-neuronal cholinergic systems represented by various tissues. It is synthesised by epithelial and endothelial cells, keratinocytes, and certain immune cells^79^. The cholinergic anti-inflammatory pathway is an important mechanism that inhibits cytokine production and minimises tissue injury during inflammation^28^. In inflammatory skin diseases, ACh exerts anti-inflammatory effects on keratinocytes, endothelial cells, fibroblasts, and macrophages^79^. Muscarinic receptors within keratinocytes modulate essential processes, including cell survival, growth, adhesion, motility, and differentiation, highlighting the regulatory role of the non-neuronal cholinergic system in epidermal homeostasis^80^. ACh has also been shown to exert an anti-fibrotic effect. In an in vitro model of corneal fibrosis, it inhibited extracellular matrix production by downregulating the expression of collagen I, III, and V, lumican, fibronectin, and α-SMA in quiescent keratinocytes. Acting on the same molecules, ACh also interrupted the transition of corneal fibroblasts into myofibroblasts after their pro-fibrotic induction^81^. In our study, we observed reductions in ACh after Zeb+RA at day 3 in 5 mm (Fig. 4a) and at day 42 in Di (Fig. 4c), with similar trends in 3- and 5-mm rings surrounding the wounds, suggesting modulation of the inflammatory response and remodelling after the epigenetic treatment. This Zeb+RA-mediated decrease in ACh on day 42 contrasts with the increase in 5-HT (Figs. 4c and 6c).

### GABA

There is scarce data in the literature on the involvement of GABA in wound healing and dermal homeostasis. Han et al.^82^ reported that GABA induced dose-dependent mitogenic activity in mouse fibroblasts in vitro and reduced the expression of inflammatory mediators, including iNOS, IL-1β, and TNF-α. In a cutaneous wound model in rats, GABA treatment accelerated wound closure, leading to full reepithelialisation and dermal fibroblast alignment. GABA also increased FGF and PDGF production in the *dermis* and *epidermis* compared to the control and, similarly to the EGF-positive control, reduced overall healing time ^82^. In a cutaneous barrier recovery model measuring transepidermal water loss (TEWL) in hairless mice, Denda et al. showed that stimulation of GABA(A) receptor in epidermal keratinocytes enhanced recovery of the barrier after topical application of GABA or GABA(A) receptor agonists—muscimol and isoguvacine compared to control^83^.

On the other hand, barrier recovery was not affected after the application of baclofen or saclofen, a GABA(B) receptor agonist and antagonist, respectively. Barrier disruption induced epidermal hyperplasia in control mice, while GABA decreased keratinocyte proliferation. The action was mediated through the GABA(A) receptor, as bicuculline methiodide, its specific antagonist, reversed the results. In our study, the overall GABA profiles were similar across treatments. Notably, however, there is a marked GABA decrease in 5-mm rings around the wounds at day 3 after Zeb+RA administration (Fig. 3a), which may indicate a modulation of the response in the acute phase after wounding.

### Final remarks

The present study is the first to profile the expression of 5-HT, 5-HIAA, GABA, ACh, and DA in wounds during ear pinna regeneration. We report an effective method for extracting neurotransmitters from solid fibrous tissues and performing HPLC-MS/MS analysis. All five examined small-molecule neurotransmitters demonstrated spatiotemporal changes in expression in regenerating tissues. Compared with normal tissues (Nt), we observed significant alterations in neurotransmitter levels in the ear pinnae on days 3 and 7 post-injury, corresponding to the inflammatory phase. Profiles of 5-HT distinguish from those of ACh, DA, and GABA. ACh, DA, and GABA tend to decrease gradually, reaching their lowest levels on day 7. In contrast, a marked increase on day 7 follows the decline in 5-HT concentrations on day 3. On day 42 post-injury, the neurotransmitters displayed even more remarkable differences compared with normal tissues (Nt). GABA displayed strong elevations in both Zeb+RA and Ctrl, whereas the effects on 5-HT and ACh were contrasting. 5-HT-decreased in Ctrl, but not in Zeb + RA, in turn, ACh declined in Zeb+RA, but not in Ctrl. 5-HIAA, a 5-HT metabolite, showed a similar reaction to that of 5-HT. While the neurotransmitters exhibited dynamic temporal changes after the injury, the spatial responses to wounding were, in principle, similar throughout the whole ear pinna. However, there were significant exceptions: 5-HT increased compared to Di in 5-mm rings around the wounds on day 7, and GABA was elevated in Di compared to the wound vicinity on day 3. Table 1 summarises key observations.

**Table 1:**
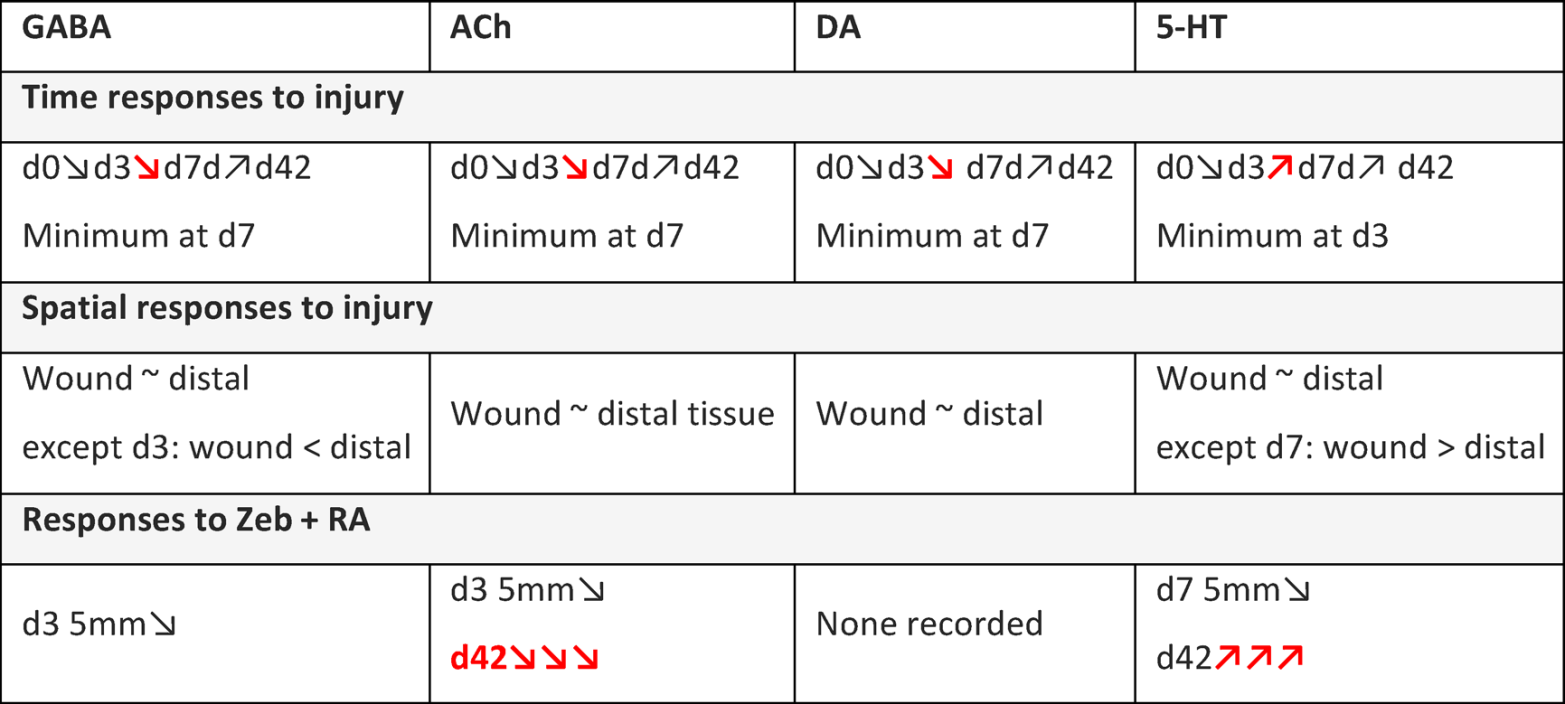
Key trends in small molecule neurotransmitter responses to tissue injury and treatment with zebularine and retinoic acid (Zeb+RA).

Symbol explanations: ~ similar to; ↘ decrease; ↗ increase.

Zeb+RA treatment, which accelerated and improved ear pinna wound closure, modulated neurotransmitter profiles. Although changes at the initial time points corresponding to the inflammatory phase seemed modest, they reached statistical significance for 5-HT, ACh, and GABA. The alterations in 5-HT and ACh levels during the remodelling phase were markedly higher. The contrasting expression of ACh and 5-HT in Zeb+RA-treated and control mice on day 42 post-injury deserves attention. Importantly, these responses could be interpreted as indirect treatment consequences, as they occurred 4 weeks after the last day of drug administration (Fig. 2a) and 3 weeks after wound closure reached its maximum degree (Fig. 2b). Typically, since ACh suppresses inflammatory cytokines and 5-HT stimulates fibrosis, low ACh and high 5-HT may be associated with prolonged inflammation and fibrotic scarring, thereby impairing regeneration. However, because we observe tissue restoration following Zeb+RA treatment, an alternative interpretation should be considered. Specifically, 5-HT elevated at the wound edges may act as a trophic factor promoting cell growth, innervation, and vascularisation, while decreased ACh prevents the suppression of tissue remodelling.

Dissecting the precise roles of small-molecule neurotransmitters in Zeb+ RA-mediated tissue regeneration is beyond the scope of the current study and requires mechanistic experiments targeting neurotransmitter pathways. However, our observations point to several interesting avenues for future analysis. First, it would be interesting to analyse whether a drop in anti-inflammatory ACh and GABA on days 3 and 5-HT on day 7, upon Zeb+RA treatment, corresponds to an accelerated initiation and, consequently, an earlier resolution of the inflammatory phase. This, in turn, raises the question of whether Zeb+RA facilitates the macrophage transition from M1 to M2 by inducing the serotonergic system during the remodelling phase. Next, it is worth investigating if high 5-HT and low ACh observed after Zeb+RA treatment during the remodelling phase may support the restoration of tissue integrity. Another potential mechanistic association between neurotransmitter expression and zebularine administration worth exploring is the previously reported activation of neurodevelopmental genes in response to the epigenetic regenerative treatment and re-innervation observed in the restored ear pinna tissues^11,74^.

## Methods

### Reagents

LC-MS grade (purity ≥ 98%) acetylcholine chloride (Sigma-Aldrich, cat. no. A6625-25G PHR1090-1G), dopamine hydrochloride (Sigma-Aldrich, cat. no. PHR1090-1G), serotonin hydrochloride (Sigma-Aldrich, cat. no. H9523-25MG), γ-Aminobutyric acid (GABA) (Sigma-Aldrich, cat. no. A2129–10G), 5-hydroxyindole acetic acid (5-HIAA) (Sigma-Aldrich, cat. no. H8876-500MG), acetonitrile (Sigma-Aldrich, cat. no. 1.00029), methanol (Avantor Performance Materials Poland S.A, cat. no. 621990110), formic acid (Avantor Performance Materials Poland S.A, cat. no. 564630116). Zebularine (cat. no. Z0022, LOT 4AL6L-TH) and retinoic acid (cat. no. R0064 LOT 75THB-MR) were purchased from TCI.

### Animals

The experiments on BALB/c (female) mice aged 8–10 weeks were conducted at the Tri-City Academic Laboratory Animal Centre of the Medical University of Gdańsk, where the animals were raised and maintained. The animal experimentation protocols were approved by the Local Ethics Committee for Animal Experimentation in Bydgoszcz, Poland (permit no. 51/2020). All experiments were performed in accordance with relevant guidelines and regulations. The study is reported in accordance with ARRIVE guidelines.

### Ear pinna punch wound and pharmacological epigenetic treatment

After anaesthesia with isoflurane by inhalation, through-and-through holes of 2 mm in diameter were made in the central part of the ear pinnae using a scissor-style ear punch (Hammacher Solingen; LOT FTC-15/8670/1). Mice were randomly divided into treatment and control groups of 6 animals each. The mice of the treatment group were injected intraperitoneally with zebularine in saline (50 mg/ml) at 500 mg/kg body weight and retinoic acid in rape seed oil (1 mg/ml) at 8 mg/kg body weight. The control mice received the vehicles alone. The administration schedule (Fig. 2a) was based on previously published data^34^ and subsequent experiments demonstrating almost complete ear pinna hole closure using twice-reduced doses of zebularine (500 mg/kg) and retinoic acid (8.0 mg/kg). Wounds were photographed weekly, and ear hole areas were quantified from the photographs using computer-assisted analysis with ImageJ^84^.

### Immunofluorescence and confocal microscopy

On days 7 and 42 post-injury, mice were sacrificed in a carbon dioxide chamber, and the ear pinnae were collected and fixed in 4% formaldehyde in PBS (10 mM, pH 7.4) at 4 °C overnight. Next, the outer aspects of the ear pinnae were peeled away and subjected to a tissue-clearing protocol, followed by immunostaining for nerve fibres. For tissue clearing, the CytoVista™ Tissue Clearing/Staining Kit (Thermo Fisher Scientific, cat. no. V11324) was used, with adjustments to incubation times and solution volumes based on outer ear pinna thickness ≤ 250 μm, as per the manufacturer’s instructions. On day 1, the outer aspects of the ear pinnae were washed in PBS twice an hour at room temperature, then submerged in Permeabilisation Buffer overnight at room temperature. On day 2, the specimens were additionally permeabilised and dehydrated by washing in increasing concentrations of methanol mixed with PBS (50%, 80%, 100%) for 8 min at room temperature each and next subjected to incubation in a bleaching solution (1 volume part of 30% hydrogen peroxide, 1 volume part of 100% DMSO, 4 volume parts of 100% methanol) overnight at 4 °C to reduce background fluorescence. On day 3, the samples were washed and rehydrated consecutively in: 20% DMSO in methanol, 80% methanol in water, 50% methanol in PBS, and PBS with 0.2% Tween™ 20 for 30 min at room temperature. Next, the specimens were incubated in Visikol HISTO Penetration Buffer for 30 min at room temperature, followed by blocking in Visikol HISTO Blocking Buffer for 1 h at 37 °C. Next, the specimens were transferred to the primary antibody—the monoclonal mouse anti-Tuj1 conjugated with Alexa Fluor-647 (Biolegend, the U.S.A., cat. no. 801201) to detect neuron-specific class III ß-tubulin prepared in Visikol HISTO Antibody Buffer and incubated overnight at 4 °C. On day 4, the ear pinnae were washed in Visikol HISTO Washing Buffer diluted in PBS five times for 20 min at room temperature, then dehydrated with increasing concentrations of methanol in PBS (50%, 80%, 100%) for 8 min at room temperature. Subsequently, the specimens were incubated in Visikol HISTO-1 Clearing solution for 10 min at room temperature, then overnight at 4 °C, and stored under the same conditions. On the day of microscopy scanning, the specimens were placed on a microscope slide, immersed in Visikol HISTO-1 Clearing solution, and covered with a glass coverslip. Microphotographs of the outer aspects of the ear pinnae were captured with the confocal microscope Zeiss LSM800 using ZEN 2.6 Software. Whole-mount ear pinna images were taken using a 10× objective lens, and a 640 nm laser was used to excite Alexa Fluor 647. Confocal scanning covered all optical slices with detectable nerve fibres. Photomicrographs were exported as TIFF files, and a maximum-intensity projection of z-stacks was generated in ImageJ to obtain single-plane images.

### Tissue collection for HPLC-MS/MS and qPCR

Tissues were collected following euthanasia of mice in a carbon dioxide chamber, with the CO_2_ concentration gradually increased to 70%. From injured and regenerating ear pinnae, 3-mm rings adjacent to the wounds (3 mm), 5-mm rings surrounding the wound area (5mm), and 2-mm discs distal to the wound (Di) were excised as shown in Fig. 1b on the final day of experiments (d3, d7, d42, Fig. 2a). Discs of 2-mm diameter excised on the day of injury (d0) served as normal tissue controls (Nt). Immediately after collection, tissues were placed in liquid nitrogen and stored at -80 °C until analysis.

### Tissue processing for HPLC-MS/MS

Samples from the right and left mouse ears were pooled, weighed (samples ranged from 5 to 10 mg) and disintegrated in 500–700 µl of cooled methanol solution in 2-ml tubes (0.1% formic acid, v/v) with a bead mill homogeniser (Elite Bead Ruptor, Omni International) using three 30-s cycles at 4 m/s with 10-s breaks with two 2-mm porcelain beads added to each sample. Next, the homogenised samples were centrifuged for 10 min at 10,000 RPM at 4 °C. The supernatants were stored at − 20 °C until the analysis.

### Assay validation

Calibration solutions were prepared for each solvent. For this purpose, 2.5 mg of the standard for each neurotransmitter was dissolved in 5 ml of methanol (0.1% formic acid, v/v). The obtained solution at 500 µg/ml was used to prepare dilutions for standard curves.

### HPLC-MS/MS

An Agilent Technologies 1260 Infinity II liquid chromatograph coupled with an Agilent Technologies 6470 Triple Quad LC/MS mass spectrometer was employed for the analysis. Analytes were separated on a Poroshell 120 EC×C18 column (4.6 × 150 mm, 2.7 μm). The injection volume was set at 10 µl. The mobile phase comprised 0.1% formic acid in water (v/v) as solvent A and acetonitrile as solvent B. Supplemental File 1 provides detailed HPLC-MS/MS conditions and recording parameters (Table S1), the elution gradient (Table S2), and performance values, including extraction recovery (Table S3), limits of quantification (LOQ), limits of detection (LOD), and linearity (Table S4).

### Gene expression quantitation

Total RNA was extracted from tissues representing individual ear pinnae using an RNeasy Mini Kit (Qiagen, Cat No. 74104). The cDNA templates were synthesised in a reaction mix consisting of 4 µl of 5x reaction buffer (250 mM Tris-HCl, 375 mM KCl, 15 mM MgCl_2_, 50 mM DTT), 100 picomoles of oligo dT_20_, 200 units of Maxima Reverse Transcriptase (ThermoScientific, cat. no. EP0742), and 80–120 ng of RNA, in a final volume of 20 µl. Real-time PCR reactions were performed in triplicates in a final volume of 10 µl containing 5 µl of FastStart Essential DNA Green Master (Roche, cat. no. 06402712001), 0.25 µl each of forward and reverse primers (10 µM), and 2 µl of cDNA on a LightCycler LC96 (Roche) using the following thermal cycling conditions: an initial denaturation at 95 °C for 600 s, followed by 38 amplification cycles consisting of denaturation at 95 °C for 10 s, annealing at 70 °C for the first 10 cycles and 60 °C for the remaining cycles for 10 s, and extension at 72 °C for 10 s. The transcript levels were calculated using the 2^− dCt^ method relative to the expression *of Tbp* and *Gapdh* reference genes. The PCR primer sequences are listed in Supplemental Table S5.

### Statistical analyses

Statistical significance was assessed using the Mann-Whitney *U* test to compare the treatment and control groups. Comparisons between ear pinna parts (Nt, 3mm, 5mm, Di) were made using the paired Friedman test, with *post hoc* pairwise comparisons conducted using the Nemenyi test. Comparisons between time points (d0, d3, d7, d42) were made using the Kruskal-Wallis test, with post hoc pairwise comparisons conducted using the Conover-Iman test and the Bonferroni correction for multiple tests. Two-sided statistics were applied, and a significance level of 0.05 was adopted. The statistical computations were performed using XLSTAT Life Sciences (Addinsoft).

### Sample size justification

Due to the pioneering nature of neurotransmitter determination in regenerating mouse ear pinna, no preliminary data were available for sample size calculation. Six animals per group were used, justified by Mead’s resources equation^85^ (E = total animals − total groups). E = 10 at a single time point (12 animals − 2 groups) meets the minimum acceptable threshold. For three time points, E = 30 was obtained. For ear pinna hole closure, a sample size of 12 (12 ear wounds from 6 mice) was selected based on previous data^34^.

## Supplementary Information

Below is the link to the electronic supplementary material.

Supplemental Fig. S1

Supplemental File 1.

Supplemental File 2

Supplemental File 3

Supplemental Table S5

## Data Availability

The analyte levels are listed in Supplemental File 2.

## References

[1] Frykberg, R. G. & Banks, J. Challenges in the treatment of chronic wounds. *Adv. Wound Care.* **4**, 560–582. 10.1089/wound.2015.0635 (2015). 10.1089/wound.2015.0635 PMC4528992 26339534

[2] Sen, C. K. et al. Human skin wounds: A major and snowballing threat to public health and the economy. *Wound Repair. Regen.* **17**, 763–771. 10.1111/j.1524-475X.2009.00543.x (2009). 19903300 10.1111/j.1524-475X.2009.00543.x PMC2810192

[3] Ackermann, P. W. & Hart, D. A. Influence of comorbidities: Neuropathy, vasculopathy, and diabetes on healing response quality. *Adv. Wound Care.* **2**, 410–421. 10.1089/wound.2012.0437 (2013). 10.1089/wound.2012.0437 PMC3842870 24688829

[4] Cheng, C. et al. Loss of innervation and axon plasticity accompanies impaired diabetic wound healing. *PLoS ONE* **8**, e75877. 10.1371/journal.pone.0075877 (2013). 24098736 10.1371/journal.pone.0075877 PMC3786937

[5] Janowska, A. et al. Atypical ulcers: Diagnosis and management. *Clin. Interv. Aging.* **14**, 2137–2143. 10.2147/cia.s231896 (2019). 31849457 10.2147/CIA.S231896 PMC6911347

[6] Falanga, V. et al. Chronic wounds. *Nat. Rev. Dis. Primers.* **8**, 50. 10.1038/s41572-022-00377-3 (2022). 35864102 10.1038/s41572-022-00377-3 PMC10352385

[7] Słonimska, P., Sachadyn, P., Zieliński, J., Skrzypski, M. & Pikuła, M. Chemotherapy-mediated complications of wound healing: An understudied side effect. *Adv. Wound Care.* **13**, 187–199. 10.1089/wound.2023.0097 (2024). 10.1089/wound.2023.0097 PMC10924052 38183626

[8] Kishi, K. *et al.* Mutual dependence of murine fetal cutaneous regeneration and peripheral nerve regeneration. *Wound Repair Regen.* **14**, 91–99, 10.1111/j.1743-6109.2005.00093.x (2006). 10.1111/j.1743-6109.2005.00093.x 16476077

[9] Mahmoud, A. I. et al. Nerves regulate cardiomyocyte proliferation and heart regeneration. *Dev. Cell.* **34**, 387–399. 10.1016/j.devcel.2015.06.017 (2015). 26256209 10.1016/j.devcel.2015.06.017 PMC4550513

[10] Buckley, G., Wong, J., Metcalfe, A. D. & Ferguson, M. W. Denervation affects regenerative responses in MRL/MpJ and repair in C57BL/6 ear wounds. *J. Anat.* **220**, 3–12. 10.1111/j.1469-7580.2011.01452.x (2012). 22066944 10.1111/j.1469-7580.2011.01452.x PMC3248659

[11] Sosnowski, P. et al. Regenerative drug discovery using Ear Pinna Punch Wound Model in mice. *Pharmaceuticals (Basel)* **15**, 610. 10.3390/ph15050610 (2022). 35631437 10.3390/ph15050610 PMC9145447

[12] Cisterna, B. A., Cardozo, C. & Sáez, J. C. Neuronal involvement in muscular atrophy. *Front. Cell. Neurosci.* **8**, 405. 10.3389/fncel.2014.00405 (2014). 25540609 10.3389/fncel.2014.00405 PMC4261799

[13] Alapure, B. V., Lu, Y., Peng, H. & Hong, S. Surgical denervation of specific cutaneous nerves impedes excisional wound healing of small animal ear pinnae. *Mol. Neurobiol.* **55**, 1236–1243. 10.1007/s12035-017-0390-0 (2018). 28110472 10.1007/s12035-017-0390-0 PMC5577384

[14] Lu, L. et al. Denervation affected skin wound healing in a modified rat model. *Int. J. Low. Extrem. Wounds* **24**, 329–341. 10.1177/15347346221090758 (2022). 35341341 10.1177/15347346221090758

[15] Nowak, N. C., Menichella, D. M., Miller, R. & Paller, A. S. Cutaneous innervation in impaired diabetic wound healing. *Transl. Res.* **236**, 87–108. 10.1016/j.trsl.2021.05.003 (2021). 34029747 10.1016/j.trsl.2021.05.003 PMC8380642

[16] Chéret, J., Lebonvallet, N., Carré, J. L., Misery, L. & Le Gall-Ianotto, C. Role of neuropeptides, neurotrophins, and neurohormones in skin wound healing. *Wound Repair. Regen.* **21**, 772–788. 10.1111/wrr.12101 (2013). 24134750 10.1111/wrr.12101

[17] Antezana, M. et al. Neutral endopeptidase activity is increased in the skin of subjects with diabetic ulcers. *J. Invest. Dermatol.* **119**, 1400–1404. 10.1046/j.1523-1747.2002.19618.x (2002). 12485446 10.1046/j.1523-1747.2002.19618.x

[18] Jain, M., LoGerfo, F. W., Guthrie, P. & Pradhan, L. Effect of hyperglycemia and neuropeptides on interleukin-8 expression and angiogenesis in dermal microvascular endothelial cells. *J. Vasc. Surg.* **53**, 1654-1660.e1652. 10.1016/j.jvs.2011.02.019 (2011). 21609799 10.1016/j.jvs.2011.02.019

[19] Scott, J. R. et al. Substance P levels and neutral endopeptidase activity in acute burn wounds and hypertrophic scar. *Plast. Reconstr. Surg.* **115**, 1095–1102. 10.1097/01.prs.0000156151.54042.da (2005). 15793451 10.1097/01.prs.0000156151.54042.da

[20] Ashrafi, M., Baguneid, M. & Bayat, A. The role of neuromediators and innervation in cutaneous wound healing. *Acta Derm. Venereol.* **96**, 587–594. 10.2340/00015555-2321 (2016). 26676806 10.2340/00015555-2321

[21] Gupta, D., Kaushik, D. & Mohan, V. Role of neurotransmitters in the regulation of cutaneous wound healing. *Exp. Brain Res.* **240**, 1649–1659. 10.1007/s00221-022-06372-0 (2022). 35488904 10.1007/s00221-022-06372-0

[22] Fan, Y. Y. et al. Nicotinic acetylcholine receptor α7 subunit is time-dependently expressed in distinct cell types during skin wound healing in mice. *Histochem. Cell Biol.* **135**, 375–387. 10.1007/s00418-011-0798-y (2011). 21437621 10.1007/s00418-011-0798-y

[23] Chakroborty, D. et al. Activation of dopamine D1 receptors in dermal fibroblasts restores vascular endothelial growth factor-a production by these cells and subsequent angiogenesis in diabetic cutaneous wound tissues. *Am. J. Pathol.* **186**, 2262–2270. 10.1016/j.ajpath.2016.05.008 (2016). 27422612 10.1016/j.ajpath.2016.05.008 PMC5012463

[24] Cooke, J. P. Angiogenesis and the role of the endothelial nicotinic acetylcholine receptor. *Life Sci.* **80**, 2347–2351. 10.1016/j.lfs.2007.01.061 (2007). 17383685 10.1016/j.lfs.2007.01.061 PMC1941778

[25] Idzko, M. et al. The serotoninergic receptors of human dendritic cells: Identification and coupling to cytokine release. *J. Immunol.* **172**, 6011–6019. 10.4049/jimmunol.172.10.6011 (2004). 15128784 10.4049/jimmunol.172.10.6011

[26] Kishibe, M., Griffin, T. M. & Radek, K. A. Keratinocyte nicotinic acetylcholine receptor activation modulates early TLR2-mediated wound healing responses. *Int. Immunopharmacol.* **29**, 63–70. 10.1016/j.intimp.2015.05.047 (2015). 26071220 10.1016/j.intimp.2015.05.047 PMC4637223

[27] León-Ponte, M., Ahern, G. P. & O’Connell, P. J. Serotonin provides an accessory signal to enhance T-cell activation by signaling through the 5-HT7 receptor. *Blood* **109**, 3139–3146. 10.1182/blood-2006-10-052787 (2007). 17158224 10.1182/blood-2006-10-052787 PMC1852236

[28] Zdanowski, R., Krzyżowska, M., Ujazdowska, D., Lewicka, A. & Lewicki, S. Role of α7 nicotinic receptor in the immune system and intracellular signaling pathways. *Cent. Eur. J. Immunol.* **40**, 373–379. 10.5114/ceji.2015.54602 (2015). 26648784 10.5114/ceji.2015.54602 PMC4655390

[29] Zoli, M., Pucci, S., Vilella, A. & Gotti, C. Neuronal and extraneuronal nicotinic acetylcholine receptors. *Curr. Neuropharmacol.* **16**, 338–349. 10.2174/1570159x15666170912110450 (2018). 28901280 10.2174/1570159X15666170912110450 PMC6018187

[30] Yamazaki, T. et al. Whole-mount adult ear skin imaging reveals defective neuro-vascular branching morphogenesis in obese and type 2 diabetic mouse models. *Sci. Rep.* **8**, 430. 10.1038/s41598-017-18581-7 (2018). 29323138 10.1038/s41598-017-18581-7 PMC5764985

[31] Lee, S. K. & Wolfe, S. W. Peripheral nerve injury and repair. *J. Am. Acad. Orthop. Surg.* **8**, 243–252. 10.5435/00124635-200007000-00005 (2000). 10951113 10.5435/00124635-200007000-00005

[32] Williams-Boyce, P. K. & Daniel, J. C. Jr. Comparison of ear tissue regeneration in mammals. *J. Anat.* **149**, 55–63 (1986). 3693110 PMC1261633

[33] Clark, L. D., Clark, R. K. & Heber-Katz, E. A new murine model for mammalian wound repair and regeneration. *Clin. Immunol. Immunopathol.* **88**, 35–45. 10.1006/clin.1998.4519 (1998). 9683548 10.1006/clin.1998.4519

[34] Sass, P. et al. Epigenetic inhibitor zebularine activates ear pinna wound closure in the mouse. *EBioMedicine* **46**, 317–329. 10.1016/j.ebiom.2019.07.010 (2019). 31303499 10.1016/j.ebiom.2019.07.010 PMC6710911

[35] Podolak-Popinigis, J., Ronowicz, A., Dmochowska, M., Jakubiak, A. & Sachadyn, P. The methylome and transcriptome of fetal skin: Implications for scarless healing. *Epigenomics* **8**, 1331–1345. 10.2217/epi-2016-0068 (2016). 27510554 10.2217/epi-2016-0068

[36] Gornikiewicz, B., Ronowicz, A., Krzeminski, M. & Sachadyn, P. Changes in gene methylation patterns in neonatal murine hearts: Implications for the regenerative potential. *BMC Genomics* **17**, 231. 10.1186/s12864-016-2545-1 (2016). 26979619 10.1186/s12864-016-2545-1 PMC4791959

[37] Gornikiewicz, B., Ronowicz, A., Madanecki, P. & Sachadyn, P. Genome-wide DNA methylation profiling of the regenerative MRL/MpJ mouse and two normal strains. *Epigenomics* **9**, 1105–1122. 10.2217/epi-2017-0009 (2017). 28758427 10.2217/epi-2017-0009

[38] Gornikiewicz, B. et al. Epigenetic basis of regeneration: Analysis of genomic DNA methylation profiles in the MRL/MpJ mouse. *DNA. Res.* **20**, 605–621. 10.1093/dnares/dst034 (2013). 23929942 10.1093/dnares/dst034 PMC3859327

[39] Shah, R. et al. Reversal of dual epigenetic repression of non-canonical Wnt-5a normalises diabetic corneal epithelial wound healing and stem cells. *Diabetologia* **66**, 1943–1958. 10.1007/s00125-023-05960-1 (2023). 37460827 10.1007/s00125-023-05960-1 PMC10474199

[40] Herranz, M. et al. The novel DNA methylation inhibitor zebularine is effective against the development of murine T-cell lymphoma. *Blood* **107**, 1174–1177. 10.1182/blood-2005-05-2033 (2006). 16239434 10.1182/blood-2005-05-2033

[41] Yilmaz, M., Kantarjian, H. & Ravandi, F. Acute promyelocytic leukemia current treatment algorithms. *Blood Cancer J.* **11**, 123. 10.1038/s41408-021-00514-3 (2021). 34193815 10.1038/s41408-021-00514-3 PMC8245494

[42] Hornung, J. P. The human raphe nuclei and the serotonergic system. *J. Chem. Neuroanat.* **26**, 331–343. 10.1016/j.jchemneu.2003.10.002 (2003). 14729135 10.1016/j.jchemneu.2003.10.002

[43] Raghupathi, R. et al. Identification of unique release kinetics of serotonin from guinea-pig and human enterochromaffin cells. *J. Physiol.* **591**, 5959–5975. 10.1113/jphysiol.2013.259796 (2013). 24099799 10.1113/jphysiol.2013.259796 PMC3872764

[44] Balaban, Z. & Kurt, G. in *Topics in Autonomic Nervous System* (IntechOpen, 2023).

[45] Mössner, R. & Lesch, K. P. Role of serotonin in the immune system and in neuroimmune interactions. *Brain Behav. Immun.* **12**, 249–271. 10.1006/brbi.1998.0532 (1998). 10080856 10.1006/brbi.1998.0532

[46] Gershon, M. D. & Tack, J. The serotonin signaling system: From basic understanding to drug development for functional GI disorders. *Gastroenterology* **132**, 397–414. 10.1053/j.gastro.2006.11.002 (2007). 17241888 10.1053/j.gastro.2006.11.002

[47] Ni, W. & Watts, S. W. 5-hydroxytryptamine in the cardiovascular system: Focus on the serotonin transporter (SERT). *Clin. Exp. Pharmacol. Physiol.* **33**, 575–583. 10.1111/j.1440-1681.2006.04410.x (2006). 16789923 10.1111/j.1440-1681.2006.04410.x

[48] Mann, D. A. & Oakley, F. Serotonin paracrine signaling in tissue fibrosis. *Biochimica et Biophysica Acta (BBA) - Molecular Basis of Disease* **1832**, 905–910. 10.1016/j.bbadis.2012.09.009 (2013). 23032152 10.1016/j.bbadis.2012.09.009 PMC3793867

[49] Lv, J. & Liu, F. The role of serotonin beyond the central nervous system during embryogenesis. *Front. Cell. Neurosci.* **11**, 74. 10.3389/fncel.2017.00074 (2017). 28348520 10.3389/fncel.2017.00074 PMC5346549

[50] de Las Casas-Engel, M. & Corbí, A. L. Serotonin modulation of macrophage polarization: Inflammation and beyond. *Adv. Exp. Med. Biol.* **824**, 89–115. 10.1007/978-3-319-07320-0_9 (2014). 25038996 10.1007/978-3-319-07320-0_9

[51] Nichols, D. E. & Nichols, C. D. Serotonin receptors. *Chem. Rev.* **108**, 1614–1641. 10.1021/cr078224o (2008). 18476671 10.1021/cr078224o

[52] Houston, D. S. & Vanhoutte, P. M. Serotonin and the vascular system. Role in health and disease, and implications for therapy. *Drugs* **31**, 149–163. 10.2165/00003495-198631020-00004 (1986). 3512233 10.2165/00003495-198631020-00004

[53] Kushnir-Sukhov, N. M. et al. 5-Hydroxytryptamine induces mast cell adhesion and migration. *J. Immunol.* **177**(9), 6422–6432. 10.4049/jimmunol.177.9.6422 (2006). 17056574 10.4049/jimmunol.177.9.6422

[54] Boehme, S. A. et al. Cutting edge: Serotonin is a chemotactic factor for eosinophils and functions additively with eotaxin. *J. Invest. Med.* **173**, 3599–3603. 10.4049/jimmunol.173.6.3599 (2004). 10.4049/jimmunol.173.6.3599 15356103

[55] Müller, T. et al. 5-Hydroxytryptamine modulates migration, cytokine and chemokine release and T-cell priming capacity of dendritic cells in vitro and in vivo. *PLoS ONE* **4**, e6453. 10.1371/journal.pone.0006453 (2009). 19649285 10.1371/journal.pone.0006453 PMC2714071

[56] Ellis, S., Lin, E. J. & Tartar, D. Immunology of wound healing. *Curr. Dermatol. Rep.* **7**, 350–358. 10.1007/s13671-018-0234-9 (2018). 30524911 10.1007/s13671-018-0234-9 PMC6244748

[57] Minutti, C. M., Knipper, J. A., Allen, J. E. & Zaiss, D. M. Tissue-specific contribution of macrophages to wound healing. *Semin. Cell Dev. Biol.* **61**, 3–11. 10.1016/j.semcdb.2016.08.006 (2017). 27521521 10.1016/j.semcdb.2016.08.006

[58] Karkanitsa, M., Fathi, P., Ngo, T. & Sadtler, K. Mobilizing endogenous repair through understanding immune reaction with biomaterials. *Front. Bioeng. Biotechnol.* **9**, 730938. 10.3389/fbioe.2021.730938 (2021). 34917594 10.3389/fbioe.2021.730938 PMC8670074

[59] Hesketh, M., Sahin, K. B., West, Z. E. & Murray, R. Z. Macrophage phenotypes regulate scar formation and chronic wound healing. *Int. J. Mol. Sci.* **18**, 1545. 10.3390/ijms18071545 (2017). 28714933 10.3390/ijms18071545 PMC5536033

[60] Koh, T. J. & DiPietro, L. A. Inflammation and wound healing: The role of the macrophage. *Expert Rev. Mol. Med.* **13**, e23. 10.1017/s1462399411001943 (2011). 21740602 10.1017/S1462399411001943 PMC3596046

[61] Oishi, Y. & Manabe, I. Macrophages in inflammation, repair and regeneration. *Int. Immunol.* **30**, 511–528. 10.1093/intimm/dxy054 (2018). 30165385 10.1093/intimm/dxy054

[62] Sadiq, A. et al. 5-HT1A receptor function makes wound healing a happier process. *Front. Pharmacol.* **9**, 1406. 10.3389/fphar.2018.01406 (2018). 30618734 10.3389/fphar.2018.01406 PMC6297675

[63] de las Casas-Engel, M. et al. Serotonin skews human macrophage polarization through HTR2B and HTR7. *J. Immunol.* **190**(5), 2301–2310. 10.4049/jimmunol.1201133 (2013). 23355731 10.4049/jimmunol.1201133

[64] Dees, C. et al. Platelet-derived serotonin links vascular disease and tissue fibrosis. *J. Exp. Med.* **208**, 961–972. 10.1084/jem.20101629 (2011). 21518801 10.1084/jem.20101629 PMC3092343

[65] Macdonald, R. A., Robbins, S. L. & Mallory, G. K. Dermal fibrosis following subcutaneous injections of serotonin creatinine sulphate. *Exp. Biol. Med.* **97**, 334–337. 10.3181/00379727-97-23734 (1958). 10.3181/00379727-97-23734 13518267

[66] Budhiraja, A. et al. Serotonin-modulating therapies for the management of chronic wounds. *Front. Pharmacol.* **16**, 1656302. 10.3389/fphar.2025.1656302 (2025). 41059203 10.3389/fphar.2025.1656302 PMC12497823

[67] Nguyen, C. M. et al. Topical fluoxetine as a novel therapeutic that improves wound healing in diabetic mice. *Diabetes* **68**, 1499–1507. 10.2337/db18-1146 (2019). 31048368 10.2337/db18-1146 PMC6609984

[68] Rodriguez-Barucg, Q. et al. Environmental fluoxetine promotes skin cell proliferation and wound healing. *Environ. Pollut.* **362**, 124952. 10.1016/j.envpol.2024.124952 (2024). 39277126 10.1016/j.envpol.2024.124952

[69] Fefeu, M. et al. Serotonin reuptake inhibitors improve muscle stem cell function and muscle regeneration in male mice. *Nat. Commun.* **15**, 6457. 10.1038/s41467-024-50220-4 (2024). 39085209 10.1038/s41467-024-50220-4 PMC11291725

[70] Li, C. et al. Could peripheral 5-HT level be used as a biomarker for depression diagnosis and treatment? A narrative minireview. *Front. Pharmacol.* **14**, 1149511. 10.3389/fphar.2023.1149511 (2023). 36959863 10.3389/fphar.2023.1149511 PMC10028199

[71] Tomasek, J. J., Gabbiani, G., Hinz, B., Chaponnier, C. & Brown, R. A. Myofibroblasts and mechano-regulation of connective tissue remodelling. *Nat. Rev. Mol. Cell Biol.* **3**, 349–363. 10.1038/nrm809 (2002). 11988769 10.1038/nrm809

[72] Guo, D. C. et al. Mutations in smooth muscle alpha-actin (ACTA2) cause coronary artery disease, stroke, and Moyamoya disease, along with thoracic aortic disease. *Am. J. Hum. Genet.* **84**, 617–627. 10.1016/j.ajhg.2009.04.007 (2009). 19409525 10.1016/j.ajhg.2009.04.007 PMC2680995

[73] McAndrews, K. M. et al. Dermal αSMA(+) myofibroblasts orchestrate skin wound repair via β1 integrin and independent of type I collagen production. *EMBO J.* **41**, e109470. 10.15252/embj.2021109470 (2022). 35212000 10.15252/embj.2021109470 PMC8982612

[74] Słonimska, P. et al. Alginate formulations with high loads of zebularine and retinoic acid promote tissue growth and innervation and induce extensive epigenetic repatterning. *Sci. Rep.* **15**, 37923. 10.1038/s41598-025-22528-8 (2025). 41162555 10.1038/s41598-025-22528-8 PMC12572142

[75] Koh, E. S. et al. The protective effect of zebularine, an inhibitor of DNA methyltransferase, on renal tubulointerstitial inflammation and fibrosis. *Int. J. Mol. Sci.* **23**, 14045. 10.3390/ijms232214045 (2022). 36430531 10.3390/ijms232214045 PMC9697081

[76] Basu, S. et al. The neurotransmitter dopamine inhibits angiogenesis induced by vascular permeability factor/vascular endothelial growth factor. *Nat. Med.* **7**, 569–574. 10.1038/87895 (2001). 11329058 10.1038/87895

[77] Shome, S. et al. Dopamine regulates angiogenesis in normal dermal wound tissues. *PLoS ONE* **6**, e25215. 10.1371/journal.pone.0025215 (2011). 21949884 10.1371/journal.pone.0025215 PMC3176820

[78] Lin, Y. C. et al. Enhancing wound healing and adhesion through dopamine-assisted gelatin-silica hybrid dressings. *Int. J. Biol. Macromol.* **258**, 128845. 10.1016/j.ijbiomac.2023.128845 (2024). 38141693 10.1016/j.ijbiomac.2023.128845

[79] Qu, H. Q., Kao, C. & Hakonarson, H. Implications of the non-neuronal cholinergic system for therapeutic interventions of inflammatory skin diseases. *Exp. Dermatol.* **33**, e15181. 10.1111/exd.15181 (2024). 39422283 10.1111/exd.15181

[80] Kurzen, H., Wessler, I., Kirkpatrick, C. J., Kawashima, K. & Grando, S. A. The non-neuronal cholinergic system of human skin. *Horm. Metab. Res.* **39**, 125–135. 10.1055/s-2007-961816 (2007). 17326008 10.1055/s-2007-961816

[81] Słoniecka, M. & Danielson, P. Acetylcholine decreases formation of myofibroblasts and excessive extracellular matrix production in an in vitro human corneal fibrosis model. *J. Cell. Mol. Med.* **24**, 4850–4862. 10.1111/jcmm.15168 (2020). 32176460 10.1111/jcmm.15168 PMC7176861

[82] Han, D., Kim, H. Y., Lee, H. J., Shim, I. & Hahm, D. H. Wound healing activity of gamma-aminobutyric acid (GABA) in rats. *J. Microbiol. Biotechnol.* **17**, 1661–1669 (2007). 18156782

[83] Denda, M., Inoue, K., Inomata, S. & Denda, S. Gamma-aminobutyric acid (A) receptor agonists accelerate cutaneous barrier recovery and prevent epidermal hyperplasia induced by barrier disruption. *J. Invest. Dermatol.* **119**, 1041–1047. 10.1046/j.1523-1747.2002.19504.x (2002). 12445190 10.1046/j.1523-1747.2002.19504.x

[84] Schneider, C. A., Rasband, W. S. & Eliceiri, K. W. NIH Image to ImageJ: 25 years of image analysis. *Nat. Methods* **9**, 671–675. 10.1038/nmeth.2089 (2012). 22930834 10.1038/nmeth.2089 PMC5554542

[85] Mead, R., Gilmour, S. G. & Mead, A. *Statistical Principles for the Design of Experiments: Applications to Real Experiments* Vol. 36 (Cambridge University Press, 2012).

