# Supplementary material for: Insights into small-molecule neurotransmitter levels and distribution during tissue regeneration in an ear pinna model in mice
Source: Sci Rep. 2026 May 20;16:23037. doi: 10.1038/s41598-026-53380-z (PMC13392258; doi:10.1038/s41598-026-53380-z)
Supplement: Supplementary file 1 — Supplemental Fig. S1 [file 41598_2026_53380_MOESM1_ESM.pdf]

Ronda et al., Insights into small-molecule neurotransmitter levels and distribution during tissue regeneration in an ear pinna model in mice, **Supplemental Figure 1.** *qPCR quantitation of Acta2 and Ccn2 transcripts*

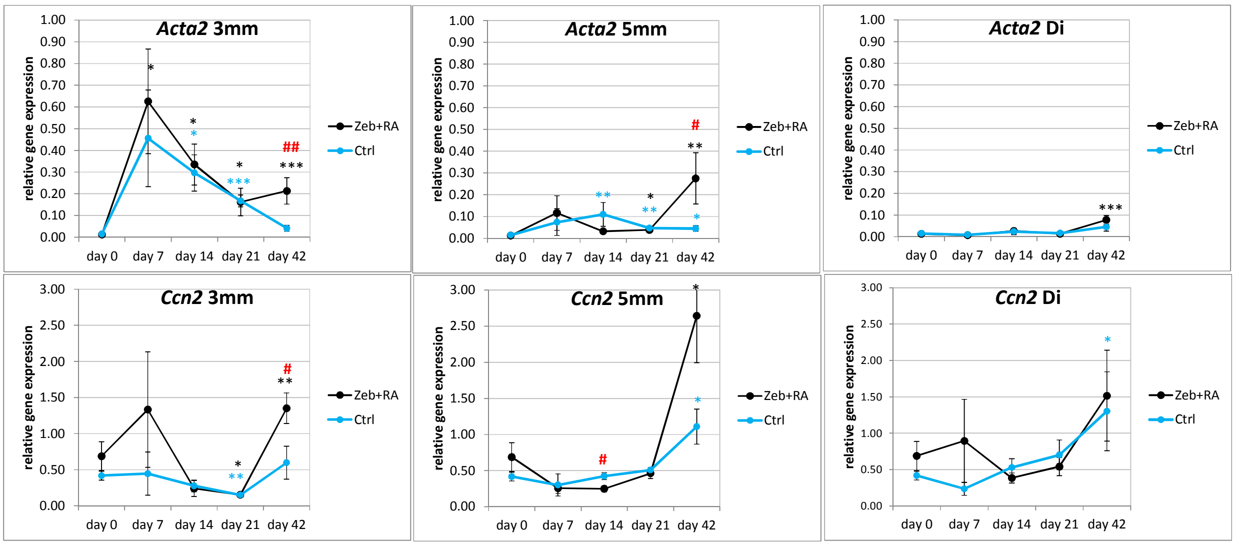

Fig. S1. Changes of fibroblast activation gene expression in regenerating ear pinna.

Acta2 (αSMA) and Ccn2 (connective tissue growth factor) expression was qPCR-determined in 3-mm and 5-m rings denoted as 3mm and 5mm) surrounding the wound and a site distant from the wound denoted as Di before injury (day 0) and on days 7, 14, 21 and 42 post-injury in mice group subjected to pharmacological treatment (Zeb+RA, n=6 wounds) and control group injected with the vehicle only Ctrl (n=6 wounds). Day 0 refers to 2-mm discs of normal tissue excised to make ear pinna holes and represents data assembled from Ctrl (n=24) and Zeb+RA mice (n=24). Error bars represent SEM. For between-treatment comparisons, a hash-tag indicates statistically significant differences of $p < 0.05$. For comparisons between day 0 and subsequent time points, blue and black asterisks indicate statistically significant differences for Ctrl and Zeb+RA groups, respectively; one, two, or three asterisks denote statistical significance of $p < 0.05$, $p < 0.01$, or $p < 0.001$. The Mann-Whitney U test was used to determine statistical significance.
