# Supplementary material for: Insights into small-molecule neurotransmitter levels and distribution during tissue regeneration in an ear pinna model in mice
Source: Sci Rep. 2026 May 20;16:23037. doi: 10.1038/s41598-026-53380-z (PMC13392258; doi:10.1038/s41598-026-53380-z)
Supplement: Supplementary file 2 — Supplemental File 1. [file 41598_2026_53380_MOESM2_ESM.pdf]

Ronda et al., Insights into small-molecule neurotransmitter levels and distribution during tissue regeneration in an ear pinna model in mice, **Supplemental File 1.** *Development of an HPLC-MS/MS method for the simultaneous determination of ACh, GABA, DOPA, 5-HT, and 5-HIAA.*

HPLC MS/MS conditions details

The analysis was conducted using high-performance liquid chromatography coupled with mass spectrometry. The study utilised the Agilent Technologies 1260 In nity II liquid chromatograph coupled with the Agilent Technologies 6470 Triple Quad LC/MS mass spectrometer. The analytes were separated on a Poroshell 120 ECxC18 column (4.6 x 150 mm; 2.7 µm). The injection volume was 10 µl. The mobile phase consisted of 0.1% formic acid in water (v/v) (solvent A) and acetonitrile (solvent B).

The elution gradient with a ow rate of 0.6 ml/min is described in Table S2. HPLC-MS/MS conditions were set as follows: gas temperature 325°C, gas ow 10 l/min, nebuliser pressure 35 psi, capillary 4000 V. Recording MS spectra for 5-HT, 5-HIAA, DA, ACh, and GABA were carried out using the multiple reaction monitoring (MRM) mode, and MS parameters speci c to each compound as listed in Supplemental File 1, Table S1.

Matrix Recovery

To calculate the matrix e ect, after sample preparation, the samples were divided into two portions, denoted as A and B. Standard solutions of neurotransmitters were added at a speci ed concentration (X) to A, and no standards were added to B. The matrix recovery (W_{matrix}) was calculated according to the formula:

$$W_{matrix} = \frac{A \quad B}{X} \quad 100\%$$

The limit of detection (LOD) was estimated for the lowest concentration of the standard solution according to the formula:

$$LOD = \frac{3.3 \quad s}{a}$$

Where: s - standard deviation; a - slope of the calibration curve.

The limit of quanti cation (LOQ) was calculated as follows:

$$LOQ = 3 \quad LOD$$

HPLC-MS/MS optimisation

The chromatographic conditions were optimised for acceptable separation, high detection sensitivity, short retention times, and well-resolved peaks. The positive ionisation mode was selected based on a literature review [58-61] and due to its higher sensitivity. The protonated molecules [M + H^+], m/z 104.1; 146.2; 148.0; 154.1; 177.1; 192.2 for GABA, ACh, DA, 5-HT, 5-HIAA, respectively, were selected as precursor ions. Collision energies were optimised using the autotune function in Agilent MassHunter software, which identi ed the most sensitive transitions and the most abundant product ions for quanti cation (Table S1). Three columns were tested for resolving power. Based on the literature data [58, 59], the rst column selected for testing was ACQUITY UPLC BEH C18 (2.1 x 100 mm, 1.7 µm). However, the resolving power of this column under the experimental conditions proved insu cient, resulting in too close retention times for di erent analytes and thus making identi cation di cult (Fig. S1a). Similar issues arose when using the column Arion Polar C18 (50 x 2.1 mm; 3.0 µm) (Fig. S1b). The next column tested, Poroshell 120 EC-C18 (4.6 x 150 mm; 2.7 µm), demonstrated satisfactory resolving power, allowing for distinct retention times for the examined neurotransmitters (Fig. S1c) and was selected for further analyses in tissue samples. The elution gradient with a ow rate of 0.6 ml/min is detailed in Table S2.

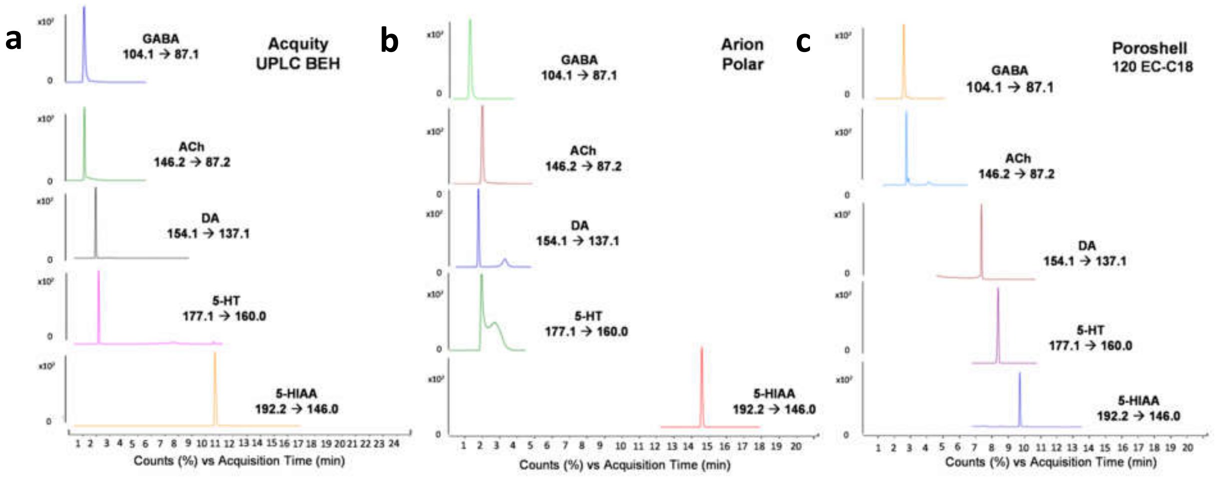

Fig S1. Chromatograms for selected neurotransmitters obtained using: ACQUITY UPLC BEH C18 (2.1 x 100 mm, 1.7 µm), Arion Polar C18 (50 x 2.1 mm; 3.0 µm) and Poroshell 120 EC-C18 (4.6 x 150 mm; 2.7 µm) columns.

Optimisation of sample preparation and extraction recovery

There are few studies on quantifying the set of small-molecule neurotransmitters selected in this study, particularly in healing wounds and regenerating solid tissues; however, data from other solid tissues, like the brain, have been reported [58-62]. Based on the literature, two solvents were selected for preliminary testing on selectivity and matrix recovery: 0.1% formic acid in methanol (v/v) and acetonitrile with 0.1% formic acid in water (2:1, v/v). The comparison revealed that 0.1% formic acid dissolved in methanol was a more suitable option, as the solvent provided satisfactory recovery for the neurotransmitters under study (Table S3).

Table S1. MS conditions for the examined neurotransmitters.

Analyte	Q1 m/z	Q3 m/z	Fragmentor	Collision energy	Polarisation	Retention time [min]
**GABA**	104.1	87.10	75	5	Positive	2.6
**ACh**	146.2	87.20	50	20	Positive	2.8
**DA**	154.1	137.1	60	5	Positive	7.6
**5-HT**	177.1	160.0	65	5	Positive	8.2
**5-HIAA**	192.2	146.0	95	20	Positive	9.8

Table S2. Elution gradient for HPLC-MS/MS

No.	Time [min]	Solvent A [%]	Solvent B [%]
**1**	2.00	100	0
**2**	8.00	50	50
**3**	10.00	10	50
**4**	11.00	10	90
**5**	12.00	100	90
**6**	13.10	100	0
**7**	21.00	100	0

Table S3. Recovery of small-molecule neurotransmitters.

	Recovery %	
**Analyte**	**0.1% formic acid in methanol**	**acetonitrile with 0.1% formic acid (2:1)**
**GABA**	103.6 ± 4.6	160.4 ± 24.9
**Ach**	97.2 ± 12.3	308.7 ± 6.2
**DA**	95.8 ± 4	28.4 ± 9.2
**5-HT**	97.8 ± 0.6	95.6 ± 1.2
**5- HIAA**	99.1 ± 1	17.8 ± 4.9

Linearity

All standards demonstrated satisfactory linearity within the concentration ranges, as shown in Table S4, although several ranges have been utilised due to high biological variation. The correlation coe cient (R) exceeded 0.99 for all analytes under the study.

Table S4. Linearity data and the validation parameters.

Neurotransmitters	The calibration curve equation	Correlation coe cient R	LOD [ng/mg]	LOQ [ng/mg]	Linear range [ng/ml]
**Parameters for analyte determination in tissues collected on day 3 post-injury**					
**GABA**	y = 2302.8x + 208.87	0.9996	0.06	0.18	0.06-1
	y = 568.25x + 2030.6	0.9998	0.384	1.150	0.39-10
**ACh**	y = 20883x + 2443.2	1.0000	0.050	0.150	0.05-5
**DA**	y = 1050.9x + 63.948	1.0000	0.032	0.100	0.03-5
**5-HT**	y = 3150.6x + 138.12	1.0000	0.011	0.030	0.01-5
**5-HIAA**	y = 831.21x + 2.4916	0.9999	0.201	0.600	0.2-10
**Parameters for analyte determination in tissues collected on day 7 post-injury**					
**GABA**	y = 4556.1x + 1650.1	1.0000	0.094	0.280	0.28-7.00
**ACh**	y = 9737.6x + 8813.1	0.9999	0.077	0.230	0.23-3.40
**DA**	y = 2953.5x + 1167.6	0.9999	0.018	0.053	0.053-0.700
**5-HT**	y = 11033x + 1472.6	0.9997	0.036	0.110	0.11-0.7
**5-HIAA**	y = 831.21x + 2.4916	0.9999	0.110	0.330	0.33-7.000
**Parameters for analyte determination in tissues collected on day 42 post-injury**					
**GABA**	y = 719.57x + 1108.4	0.9998	0.190	0.570	0.190-5
	y = 1009.5x - 1194.8	0.9997	1.512	4.540	1.512-50
**ACh**	y = 10369x + 14729	1.0000	0.020	0.050	0.020-5
	y = 9549.4x + 14412	1.0000	0.395	1.190	0.395-50
**DA**	y = 143.56x + 4.2985	1.0000	0.015	0.040	0.015-5
	y = 190.99x - 115.15	0.9996	2.841	8.520	2.841-50
**5-HT**	y = 2924x + 204.2	0.9999	0.058	0.170	0.058-5
	y = 3456.3x - 6362	0.9999	1.181	3.540	1.181-50
**5-HIAA**	y = 2924x + 204.2	0.9999	0.061	0.180	0.061-5
	y = 268.94x - 465.75	1.0000	0.662	1.980	0.662-50
