# Supplementary material for: Insights into small-molecule neurotransmitter levels and distribution during tissue regeneration in an ear pinna model in mice
Source: Sci Rep. 2026 May 20;16:23037. doi: 10.1038/s41598-026-53380-z (PMC13392258; doi:10.1038/s41598-026-53380-z)
Supplement: Supplementary file 5 — Supplemental Table S5 [file 41598_2026_53380_MOESM5_ESM.pdf]

Ronda et al., Insights into small-molecule neurotransmitter levels and distribution during tissue regeneration in an ear pinna model in mice, **Supplemental Table S5.** *PCR primers*

Table S1. PCR primer sequences

**Gene**	**Forward primer**	**Reverse primer**	**Amplicon size [bp]**
Acta2	AAGAGCTACGAACTGCCTGACG	GTTTCGTGGATGCCCGCTGA	119
Ccn2	TGAGGCTGAGTCCAGCTGTTCTTT	ACTTGCCACAAGCTGTCCAGTCTA	118
Tbp	GAGAGCCACGGACAACTGCG	GGGAACTTCACATCACAGCTC	187
Gapdh	TGGCCTTCCGTGTTCCTAC	GAGTTGCTGTTGAAGTCGCA	178
